# Satellitome landscape analysis of *Megaleporinus macrocephalus* (Teleostei, Anostomidae) reveals intense accumulation of satellite sequences on the heteromorphic sex chromosome

**DOI:** 10.1038/s41598-019-42383-8

**Published:** 2019-04-10

**Authors:** Ricardo Utsunomia, Duílio Mazzoni Zerbinato de Andrade Silva, Francisco J. Ruiz-Ruano, Caio Augusto Gomes Goes, Silvana Melo, Lucas Peres Ramos, Claudio Oliveira, Fábio Porto-Foresti, Fausto Foresti, Diogo Teruo Hashimoto

**Affiliations:** 1 Departamento de Morfologia, Instituto de Biociências, Universidade Estadual Paulista - UNESP, Distrito de Rubião Junior, s/n, 18618-970 Botucatu, SP Brazil; 2 Departamento de Genética, Universidad de Granada, 18071 Granada, Spain; 3 Departamento de Ciências Biológicas, Faculdade de Ciências, Universidade Estadual Paulista - UNESP, Campus de Bauru, 17033-360 Bauru, SP Brazil; 4 CAUNESP, Universidade Estadual Paulista - UNESP, Campus Jaboticabal, 14884-900 Jaboticabal, SP Brazil

## Abstract

The accumulation of repetitive DNA sequences on the sex-limited W or Y chromosomes is a well-known process that is likely triggered by the suppression of recombination between the sex chromosomes, which leads to major differences in their sizes and genetic content. Here, we report an analysis conducted on the satellitome of *Megaleporinus macrocephalus* that focuses specifically on the satDNAs that have been shown to have higher abundances in females and are putatively located on the W chromosome in this species. We characterized 164 satellite families in *M*. *macrocephalus*, which is, by far, the most satellite-rich species discovered to date. Subsequently, we mapped 30 satellites, 22 of which were located on the W chromosome, and 14 were shown to exist only on the W chromosome. Finally, we report two simple, quick and reliable methods that can be used for sex identification in *M*. *macrocephalus* individuals using fin clips or scales, which could be applicable to future studies conducted in the field of aquaculture.

## Introduction

Eukaryotic genomes have a large number of repetitive DNA sequences that include transposable elements (TEs), multigene families and satellite DNAs (satDNAs)^1^. These sequences are highly dynamic at the chromosomal and nucleotide levels and several mechanisms that contribute to their expansion/contraction are known to shape their evolution^2^. Satellite DNAs are noncoding head-to-tail tandemly repeated sequences that constitute long arrays that are preferentially distributed within pericentromeric or subtelomeric heterochromatic areas; however, several examples of short arrays that are dispersed within euchromatin have already been reported^3–7^. In general, groups of related species share several satDNA families that usually evolve independently within each lineage, according to the so-called library hypothesis^8^.

The use of next generation sequencing (NGS) data and bioinformatics to identify repetitive sequences provides a unique opportunity to characterize large collections of satellite DNAs in nonmodel species and, most importantly, compare satellitomes or specific satDNAs from distinct species^9–11^. In this context, the analysis of repeat diversity and genomic abundance in different species becomes possible and also allows for the isolation and characterization of some satDNAs that have accumulated within specific genomic regions, such as the B or sex chromosomes^9,12^.

Heteromorphic sex chromosomes usually arise from a loss of recombination on sex-limited W or Y chromosomes, which leads to degenerative processes such as the pseudogenization and invasion of repetitive DNA sequences^13^. Unlike mammals and birds, fishes exhibit a rapid turnover in their sex chromosomes that is directly related to the numerous sex chromosome systems described for their group^14^. Although knowledge regarding the molecular mechanisms underlying sex determination in fishes is still limited, the potential role of the accumulation of DNA repeats during sex chromosome differentiation has been well documented^13,15^. Thus, an initial mass characterization of accumulated repeats in the sex chromosomes of a particular species is necessary for better understanding of the evolution of these chromosomes, as well as to provide support for the complete assembly of repeat-rich genomic regions^2,16^.

Sex-specific markers are important noninvasive tools that can be used to assess the sex of individuals at any stage of life. Over the past years, distinct sex markers have been successfully identified for several aquatic species using a number of approaches, including amplified fragment length polymorphism (AFLP)^17,18^, random amplified polymorphic DNA (RAPD)^19,20^, and, more recently, restriction-associated DNA sequencing (RADseq) and its variations (such as ddRADseq, 2b-RAD, and GBS)^21–23^. Such markers are important for revealing sex chromosome systems and their evolution in primitive vertebrate species, as well as for providing insights into topics relevant to practical aquaculture, such as precocious sex identification, especially in species lacking morphological sexual dimorphism. They are also necessary for the identification of the genetic sex of sex-reversed individuals after hormonal and/or temperature-based treatments^14,24^.

*Megaleporinus* is a fish genus within Anostomidae that is composed by 16 species, based on morphological, molecular and cytogenetic data^25^. Interestingly, representatives of this genus share an ancestral, highly differentiated ZZ/ZW sex chromosome system^25^ and comprise the only fish genus relevant to Brazilian aquaculture that has heteromorphic sex chromosomes. Although this sex chromosome system was described more than 30 years ago^26^, our knowledge of the molecular structures and sequence components of the Z and W chromosomes is essentially restricted to the identification of the conserved presence of a dispersed repetitive element known as *LespeI* in the W chromosome in several *Megaleporinus* species^27–29^.

The species *M*. *macrocephalus*, which is popularly known as ‘piauçu’, is a valuable characiform fish that has been intensively cultivated in Brazil, with production rates reaching 3,800 tons per year^30^. Similarly, to other Brazilian native fish species, females exhibit higher growth rates than males^31^, which has led to an interest in producing superfemale (WW) specimens and, consequently, all-female broods. Thus, expanding the knowledge of the composition of the sex chromosomes of *M*. *macrocephalus* is the first step that is necessary to maximize the production of this species in the future. Additionally, the development of noninvasive tools that can be used for sexing *M*. *macrocephalus* individuals is also important to avoid sex bias in the selected population; for example, obtaining chromosomes from each specimen would be laborious and demanding, at least in juvenile member of the species. In this context, we characterized the satellitome of *M*. *macrocephalus* by integrating genomic and chromosomal data, with a special focus on those satellites that exhibit higher abundances within the female genomic library and, therefore, are putatively located on the W chromosome; the aim of this is to provide a starting point for a detailed and comprehensive analysis of the sex chromosome system. We then mapped the same satDNAs in *Megaleporinus obtusidens*, a species that diverged from *M*. *macrocephalus* approximately 9.27 mya^25^, in order to investigate the conservation of satDNA repeats in the W chromosomes of both species. Finally, since sexing individuals of this species is not possible unless mitotic chromosomes were obtained, we developed noninvasive methods for sexing multiple individuals of *M*. *macrocephalus* that utilizes a novel quick-FISH protocol and qPCR, which will be useful for future studies and in aquaculture.

## Material and Methods

### Material, DNA extraction and chromosome preparation

Live specimens of *M*. *macrocephalus* and *M*. o*btusidens* were collected from fish tanks at the Aquaculture Center of São Paulo State University, CAUNESP, Jaboticabal, SP. The specimens were anesthetized, and total blood was collected from the caudal vein, and then the individuals were returned to the fish tanks at CAUNESP. The procedures used for the sampling, maintenance and analysis of the fishes were performed in compliance with the standards of the Brazilian College of Animal Experimentation (COBEA) and approved (protocol 1094/2018) by the Bioscience Institute/UNESP Ethics Committee on the Use of Animals (CEUA).

One female and one male specimen of *M*. *macrocephalus* were selected for next generation sequencing (NGS), and the blood collected from both individuals was stored in 95% ethanol. Genomic DNA extraction from the total blood was performed using the DNeasy kit (Qiagen) according to the manufacturer’s instructions and included a RNA removal step that utilized RNAse A (Invitrogen). To obtain the cell suspensions that were used for the chromosome preparations for the *M*. *macrocephalus* and *M*. o*btusidens* specimens, we performed lymphocyte culture experiments in 12 individuals of *M*. *macrocephalus* (9 males and 3 females) and 3 individuals of *M*. *obtusidens* (3 females) according to the protocol described by Fenocchio and Bertollo^32^. Subsequently, we performed a random sampling of 20 individuals from among the offspring of *M*. *macrocephalus* for testing of our noninvasive tools. In total, we sampled two, twelve and twenty specimens for the purposes of NGS, lymphocyte culture and the diagnosis of sex, respectively.

### Whole-genome sequencing and satellitome characterization

Genomic DNA sequencing was performed on the Illumina MiSeq platform (2 × 250 bp paired-end), which yielded 0.77 Gb for the female (ZW) and 0.375 Gb for the male (ZZ) (approximately 0.5x and 0.25x genome coverage, respectively)^33^. We deposited the two libraries into the Sequence Read Archive (SRA) database under accession numbers SRR7263033 and SRR7263034 for the male and the female, respectively.

To perform a high-throughput analysis of the satellite DNA within the *M*. *macrocephalus* genome, we used the satMiner bioinformatic protocol as described by Ruiz-Ruano *et al*.^10^. Briefly, we performed quality trimming of the female and male gDNA libraries using Trimmomatic software (options used: ILLUMINACLIP:TruSeq. 3-PE.fa:2:30:10 LEADING:3 TRAILING:3 SLIDINGWINDOW:4:20 MINLEN:250) to select pairs of reads for which Q > 20 for all nucleotides^34^. We then randomly selected 2 × 200,000 reads from the female and the male and joined both selections. We performed a clustering of these 2 × 400,000 reads using RepeatExplorer^9^ with the default options to select clusters with a structure typical of satDNA (e.g., spherical or ring-shaped), and searched for contigs with tandem repetitions using the dotplot tool that is integrated into Geneious 8.1 software (Biomatters). The assembled contigs of all the clusters identified by RepeatExplorer were filtered using DeconSeq software^35^ and the remaining sequences were clustered using RepeatExplorer, which duplicated the number of reads for the second round (2 × 400,000 reads for the female and the male) to generate a total of 2 × 800,000 reads. We repeated these clustering and filtering steps until no new satDNA sequences appeared, while maintaining the number of reads during each iteration.

After satDNA mining, we performed a homology search of all the repeated unit sequences that were found and grouped them into the same sequence variant, family and superfamily if their identity was greater than 95%, 80% and 40%, respectively. We determined the abundance and divergence of each variant in the male and female libraries using the RepeatMasker software^36^ with the *cross_match* search engine and a selection of 1,494,006 reads per genome, which corresponded to the number of reads in the smallest library (male) after trimming. We estimated the abundance of each satDNA in the male (ZZ) and female (ZW) genomes based on the proportion of the aligned nucleotides within the number of total reads. Subsequently, we sorted the satDNA families found in the female genome in decreasing order of abundance and assigned a catalog number to each satDNA family according to the criteria described by Ruiz-Ruano *et al*.^10^. The consensus sequences for each satDNA family were deposited in GenBank under the accession numbers MG818994-MG819157. We also estimated the average divergence generated within a repeat landscape by considering the distances between sequences based on the Kimura 2-parameter model using the script calcDivergenceFromAlign.pl within the RepeatMasker suite^36^. Thus, by comparing the abundance and divergence of different subclasses of satellite DNAs in males and females, we constructed a subtractive repeat landscape that reveals the elements with the greatest differences in abundance in the two types of libraries, which provided the first indication of which satDNA sequences are overrepresented in the female library with respect to the male library. Finally, we calculated the quotient between the female and male abundance values of each satDNA (F/M ratio) in order to reveal the differences in abundance that may be due to differences in the autosomes and sex chromosomes. Thus, to identify potential satDNAs that are clustered on the W chromosome, we selected the 31 satDNA families that had the highest F/M ratios. Primers were then manually designed for each orientation with similar melting temperatures and less stable extensive dimers based on predictions made by the software PerlPrimer^37^ to amplify the satDNAs (Supplementary Table S2). We designed primers for all selected satDNAs, with the exception of MmaSat148, because it contained a deletion with respect to the remaining members of SF13 and there were no available regions that could anchor a pair of primers. As a result, we designed primer pairs for 30 satDNA families in total.

Statistical analysis was performed using the Shapiro-Wilks test to ascertain whether the variables fit a normal distribution, as well as the nonparametric Mann-Whitney U test, using Statistica 6.0 software.

### Retrieving of monomers from raw reads and intragenomic analysis

We collected monomers from four satDNAs families clustered on the W chromosome (MmaSat9, MmaSat97, MmaSat122 and MmaSat12) to obtain reliable scores for the haplotype abundances of these four satDNAs. For this purpose, we used a selection of 1,494,006 reads each from the female and male genomes and aligned these reads against each of the abovementioned satDNAs using with RepeatMasker to trim the ends to obtain the full monomers as described in Utsunomia *et al*.^11^. Subsequently, the collected monomers were aligned separately using the MUSCLE algorithm^38^ with the default parameters, and singletons (e.g., sequence variants found only once) were discarded at this stage. Finally, we constructed minimum spanning trees (MSTs) for each satDNA on the basis of the pairwise differences and consideration of the relative abundances of the haplotypes using PHYLOViZ 2.0 software^39^.

### FISH analysis for chromosomic satDNA mapping

Prior to the FISH experiments, all probes were labeled with digoxigenin-11-dUTP via PCR. FISH was performed in highly stringent conditions using the method described by Pinkel *et al*.^40^ with small modifications that were described in Utsunomia *et al*.^11^. Chromosomal preparations from lymphocyte cultures were counterstained with DAPI (4′,6-diamino-2-phenylindole, Vector Laboratories) and analyzed using an optical microscope (Olympus BX61). The images were captured using Image Pro Plus 6.0 software (Media Cybernetics). A minimum of 10 cells from each individual were analyzed to confirm the FISH results.

### Use of quick-FISH and qPCR for non-invasive sexing

A novel quick-FISH method was employed to develop a rapid and noninvasive tool for sexing live specimens of *M*. *macrocephalus*. We used a mixture of probes that were labeled with digoxigenin-11-dUTP that corresponded to two satDNAs: i) MmaSat97-39, a satDNA that maps to a single band that is exclusive to the W chromosome that can be used to distinguish males and females; ii) MmaSat98-37, a satDNA that maps to a single band in an autosomal pair that shows no differences between males and females and that was used as positive control for FISH. The results of FISH in interphasic cells using this combination of probes showed two spots in males, which corresponded to one per autosome of the pair containing MmaSat98-37, and three spots in females, due to the presence of the additional band corresponding to MmaSat97-39 in the W chromosome. To verify its reliability, we performed the experiment using 20 live specimens that were not subject to previous sexing. For each specimen, we extracted a single scale and a small piece of caudal fin and fixed this material directly in 200 μL of Carnoy’s solution. Subsequently, 20 μL from each cell suspension was placed onto a slide and dried for 15 min at 60 °C. We then proceeded with the FISH protocol as described above using 15 min of probe hybridization. The post-hybridization washes were similar to those performed during “regular” FISH. For each specimen, we counted between 40 to 90 cells. After the scale and fin collection, metaphasic chromosomes were obtained from the specimens using the protocol described in Foresti *et al*.^41^ in order to confirm the presence/absence of the W chromosome.

The determinations of the comparative relative abundances of the satDNAs in the male and female genomes was performed using qPCR to measure the copy number differences between males and females in both species. We selected MmaSat97 and MmaSat98 for analysis, since both were used with the quick-FISH protocol to confirm the FISH and bioinformatics results and because these may provide an additional noninvasive tool for sexing individuals. For this purpose, we selected three individuals of each sex (previously genotyped via karyotyping) from *M*. *macrocephalus* and *M*. *obtusidens*. Since the selected satDNAs are clustered in both species (Supplementary Fig. S1), this tool would be useful for both of them. qPCR was used to determine the satDNA dose using the ΔCt method of relative quantification (RQ), with the single-copy gene *hypoxanthine phosphoribosyltransferase* (Hprt) being utilized as a reference. Amplification was performed with the following primers: HprtF: 5′-GGCCAGGGAGATCATGAAGG-3′ and HprtR: 5′-TGGAGCGGTCACTATTTCGG-3′. This primer pair was designed based on the assembly of Hprt gene of *Astyanax paranae* (Silva *et al*., unpublished). The qPCR was performed using a StepOne Real-Time PCR System (Life Technologies, Carlsbad, CA). The target and reference sequences were simultaneously analyzed using two independent replicates. After amplification, we analyzed the melting curves to verify the presence of a unique product of amplification. The results from samples with inconsistent Ct values were discarded. We determined the ΔCt values, which are represented as the mean ± standard error of the mean (SEM). Statistical analysis was performed using the Shapiro-Wilks test to ascertain whether the variables fit a normal distribution, followed by the nonparametric Kruskal-Wallis test and the Student-Newman-Keuls test.

## Results

### The W chromosome of *M*. *macrocephalus* is enriched in satDNA

Based on 10 iterations of the satMiner protocol, we assembled 514 satDNA variants that belonged to 164 satDNA families that had repeat unit lengths (RUL) that ranged from 5 to 1969 bp (median value 54 bp; Supplementary Table S1 and Fig. S2). In total, satellite DNAs represented 13.47% (female) and 11.99% (male) of the genome, with 2.78% being represented by the most abundant repeat and 1.8E-08% being represented by the less abundant repeat (Supplementary Table S1). The distribution of the lengths was biased due to a predominance of short satellites, with more than half (107) being shorter than 100 bp. The A + T content of the consensus satDNA sequences varied between 29.7% and 84.2% among the families, with a median value of 58.3%, which indicated a slight bias towards A + T rich satellites. The Shapiro-Wilks test showed that the A + T content was the only satellitome feature that fit a normal distribution (W = 0.98, P = 0.08), while the remaining variables (RUL, abundance and divergence) were not normal (P < 0.05 in all cases). For this reason, we used nonparametric tests for the subsequent analysis. Short (<100 bp) and long (>100 bp) satellites had a similar amount of A + T content (U = 2710, P = 0.242). In addition, long satDNAs were more abundant in males (U = 2205, P = 0.007) but were also less diverse (U = 1474, P = 0.001). Sequence comparison between all satDNA families revealed some homology between several of them (Supplementary Table S1, Supplementary Fig. S3). Notably, we found that some long satDNAs shared a conserved motif (68–73 bp), but no evidence of a common origin among them was found (Supplementary Fig. S1). Evidence of higher order repeats (HORs) were found in some members of SF2, including MmaSat19, MmaSat20, MmaSat39, MmaSat66 and MmaSat90 (Supplementary Fig. S4).

Several satDNA families were more abundant within the female (ZW) library (Supplementary Fig. S5). Among the 164 satDNA families, we found 95 satDNAs with an F/M ratio higher than 1 and 65 satDNAs with an F/M ratio lower than 1. In addition, three satellites were not found within the male library (Table 1 and Supplementary Table S1). The subtractive repeat landscape revealed a high proportion of MmaSat1 in the female library (Supplementary Fig. S5c); however, we did not design primers for this sequence, since there were several other satDNAs with a higher (F/M) ratio (Supplementary Table S1). This could also be related to the fact that MmaSat1 is likely spread over several autosomes.

**Table 1:** Repeat unit lengths (RULs, in nt), A + T content (%), number of variants (V), and abundances (% of the libraries) in females (F) and males (M); divergence (%) in females (F) and males (M); clustering patterns and chromosomal locations of the 31 satDNAs families and superfamilies (SF) showing the highest F/M ratio (Females/Males) values.

SF	satDNA family	RUL	A + T	V	Abundance (%)		Divergence (%)		(F/M)	Pattern	Location
					F	M	F	M			
15	MmaSat155-71	71	50.7	1	0.000152		15.94		—	B	W(i)
13	MmaSat158-39	39	41	1	0.000093		26.05		—	D	W(p,i)
	MmaSat162-48	48	47.9	1	0.000003		35.77		—	D	W(i)
	MmaSat036-74	74	62.2	1	0.068342	0.000202	6.42	26.04	338.5455746	B	W(i)
	MmaSat097-39	39	53.8	3	0.024361	0.000274	10.39	14.26	88.86575875	B	W(i)
	MmaSat122-54	54	57.4	6	0.013087	0.000266	9.96	16.68	49.1743487	B	W(p,i)
13	MmaSat063-47	47	42.6	7	0.041342	0.000943	12.26	20.11	43.81854155	B	W(i)
4	MmaSat139-47	47	70.2	1	0.005029	0.000126	20.27	20.93	39.95338983	B	W(i)
	MmaSat128-38	38	57.9	4	0.011009	0.000373	9.47	15.14	29.52932761	B	W(p)
	MmaSat127-42	42	64.3	6	0.011488	0.000682	10.8	25.76	16.8478686	B	W(p)
4	MmaSat113-52	52	61.5	5	0.016611	0.001260	12.05	18.25	13.18306878	B	W(i)
	MmaSat092-46	46	54.3	11	0.028944	0.003685	14.74	33.82	7.853690304	B	W(i)
15	MmaSat058-71	71	49.3	3	0.044410	0.006081	9.57	9.95	7.303293426	DB	W(p) + A
7	MmaSat153-40	40	65	1	0.000377	0.000073	26.6	15.54	5.141818182	NS	—
8	MmaSat061-33	33	45.5	1	0.042897	0.008779	10.86	12.19	4.886482382	DB	W(d) + A
8	MmaSat151-33	33	48.5	1	0.000646	0.000158	26.07	32.66	4.086003373	NS	—
	MmaSat111-33	33	57.6	3	0.017022	0.004862	16.72	30.69	3.501042124	B	W(i)
12	MmaSat145-67	67	55.2	1	0.001107	0.000370	10.57	10.84	2.994227994	DB	W(d) + A
	MmaSat017-72	72	57.9	6	0.128001	0.056303	9.01	9.7	2.273440312	DB	W(i,d) + A
8	MmaSat150-31	31	58.1	1	0.000685	0.000319	15.86	21.31	2.147157191	B	W(p)
	MmaSat152-31	31	61.3	1	0.000413	0.000211	29.91	31.53	1.957016435	DB	W(i) + A
1	MmaSat009-53	53	58.5	5	0.251590	0.137215	9.15	10.23	1.833544197	DB	W(d) + A
	MmaSat099-31	31	61.3	7	0.023434	0.013165	10.89	11.75	1.779963135	DB	W(i) + A
	MmaSat154-30	30	43.3	1	0.000305	0.000181	25.44	32.57	1.685840708	NS	—
	MmaSat029-32	32	46.9	8	0.084698	0.050374	14.12	15.34	1.68137639	D	W(i) + A
	MmaSat107-44	44	54.6	1	0.017750	0.010899	5.84	7.03	1.628611974	DB	A
	MmaSat098-37	37	45.9	7	0.024099	0.014946	13.69	15.41	1.61236797	B	A
	MmaSat048-1298	1298	49.2	1	0.058376	0.036578	6.3	7.51	1.595953778	B	A
5	MmaSat118-66	66	68.2	2	0.015043	0.009519	9.16	10.05	1.580305365	NS	—
2	MmaSat108-295	295	60.3	1	0.017656	0.011712	6.92	9.63	1.507559543	B	A

For each family, the length and A + T content are shown for the most abundant variant. Divergence per family is expressed as a percentage of the Kimura divergence. We designed primers, produced probes and performed FISH for all variants. Pattern: B = banded, NS = no signal, D = dotted, DB = dotted-banded (based on the criteria described by Ruiz-Ruano *et al*. (2018). Chromosomal location: W = W chromosome, A = autosome, i = interstitial, p = proximal to the centromere, d = distal. When a satDNA was present at two loci in a same chromosome, both locations are indicated and separated by a comma. satDNA families are listed in decreasing order of F/M ratios.

### Chromosomal mapping reveals 14 satDNAs that are exclusive to the W chromosome

**Figure 1:**
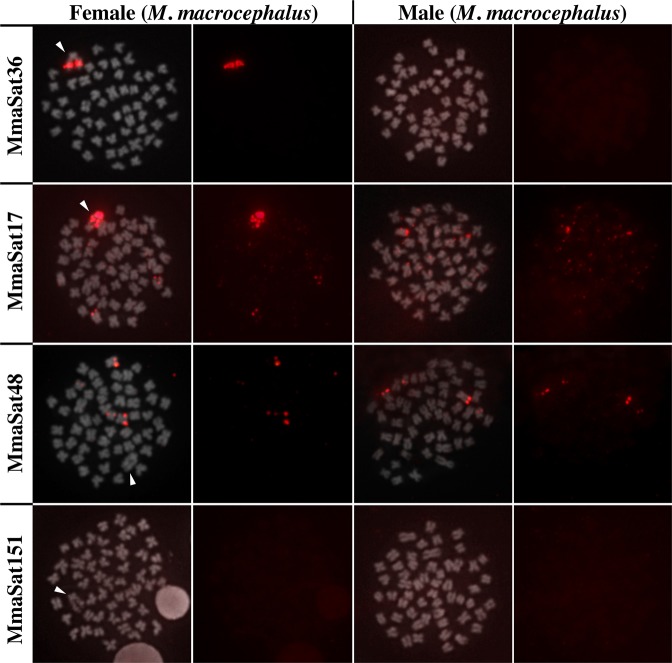
Examples of the chromosomal distribution patterns of the mapped satDNAs in *M*. *macrocephalus*; those clustered solely on the W chromosome (MmaSat36); those clustered on the W chromosome and some autosomes (MmaSat17); those clustered on the autosomes (MmaSat48); and those that are nonclustered (MmaSat151). Each cell is shown with the satDNA FISH signal (red) merged with that of DAPI (left) and satDNA FISH (right). Arrowheads indicate the W chromosome. Bar = 10 μm.

**Figure 2:**
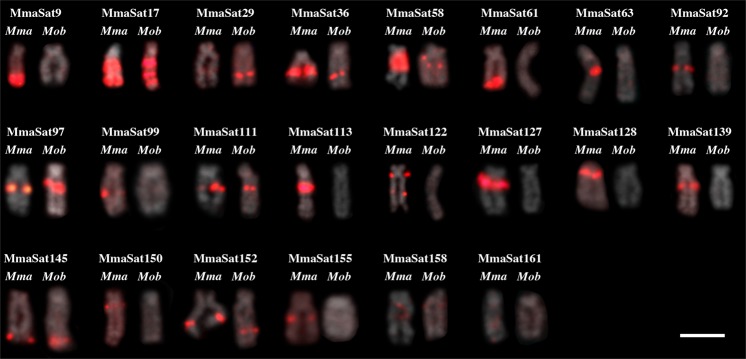
FISH analysis showing several satDNA families on the W chromosomes of *M*. *macrocephalus* (Mma) and *M*. *obtusidens* (Mob). Bar = 10 μm.

Within the 30 satDNA families that were analyzed using FISH, 26 contained conspicuous clusters in at least one chromosome (clustered pattern) in the female specimens of *M*. *macrocephalus*, while four satDNAs (MmaSat114, MmaSat151, MmaSat153 and MmaSat154) were not clustered (Table 1 and Supplementary Fig. S1). Based on the resolution of the FISH analysis, 14 were mapped exclusively to the W chromosome, 8 were located on the W chromosome and some autosomes, and 4 were clustered on autosomes (Figs 1–2 and Supplementary Fig. S1). These same probes were hybridized against *M*. *obtusidens* chromosomes, 19 of which were shown to be clustered while 11 showed a nonclustered organization (Supplementary Fig. S1). In total, for *M*. *obtusidens*, 4 satDNAs were mapped exclusively to the W chromosome, 7 were located on the W chromosome and some autosomes, and 8 were located on autosomes (Fig. 2 and Supplementary Fig. S1). Finally, the W chromosomes of both species were shown to share 10 satDNAs (Fig. 2).

Different homogenization patterns were observed in males and females. When the satellite DNA was clustered in both males and females, the satDNA was homogenous in both sexes; however, those satDNAs which clustered solely on the W chromosome had higher homogenization rates than those that were nonclustered in males, which allowed us to infer that homogenization of repeats is higher in clustered repeats (Table 1; U = 20, P = 0.003).

### Amplification and diversification of satDNA copies on the W chromosome

**Figure 3:**
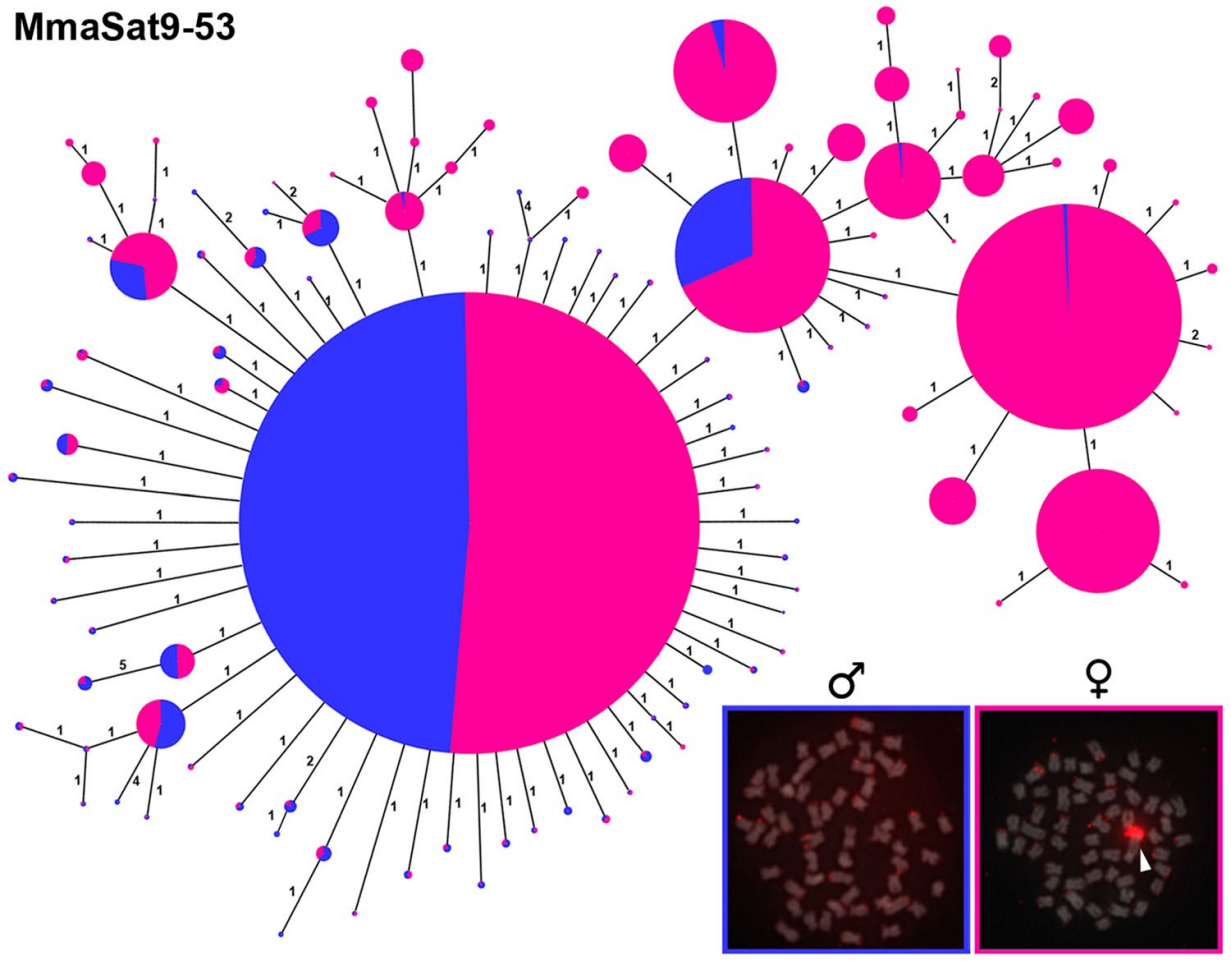
Minimum spanning tree (MST) showing the relationships between the different haplotypes of MmaSat9 that obtained from Illumina reads from males (blue) and females (pink). The diameter of the circles is proportional to their abundance and the numbers represent the number of mutational steps. Metaphasic plates after FISH of MmaSat9 in female and male specimens are shown; the colors of the borders correspond to the colors of the circles.

We successfully extracted monomers directly from Illumina raw reads representing the MmaSat9, MmaSat97, MmaSat122 and MmaSat128 satDNA families in *M*. *macrocephalus* and, after discarding singletons, we obtained 2162 monomers (110 haplotypes), 182 monomers (28 haplotypes), 152 monomers (91 haplotypes) and 141 monomers (9 haplotypes), respectively, for each family (Fig. 3 and Supplementary Fig. S6). The obtained MSTs were different for each satDNA. The MST for MmaSat9 exhibited an interesting pattern of satDNA organization, with the presence of several shared haplotypes in the males and females, which were probably located on the autosomes, as well as some haplotypes that were restricted to females, which were probably located on the W chromosome (Fig. 3). For the other satellites, a few monomers were retrieved from the male library (Supplementary Fig. S6). For MmaSat122, significant diversification in the monomers from the female was observed, while the monomers representing MmaSat128 exhibited less variation and an intermediate level of variation was observed for MmaSat97 (Supplementary Fig. S6).

### Development of two noninvasive tools for sexing individuals based on quick-FISH and qPCR

**Figure 4:**
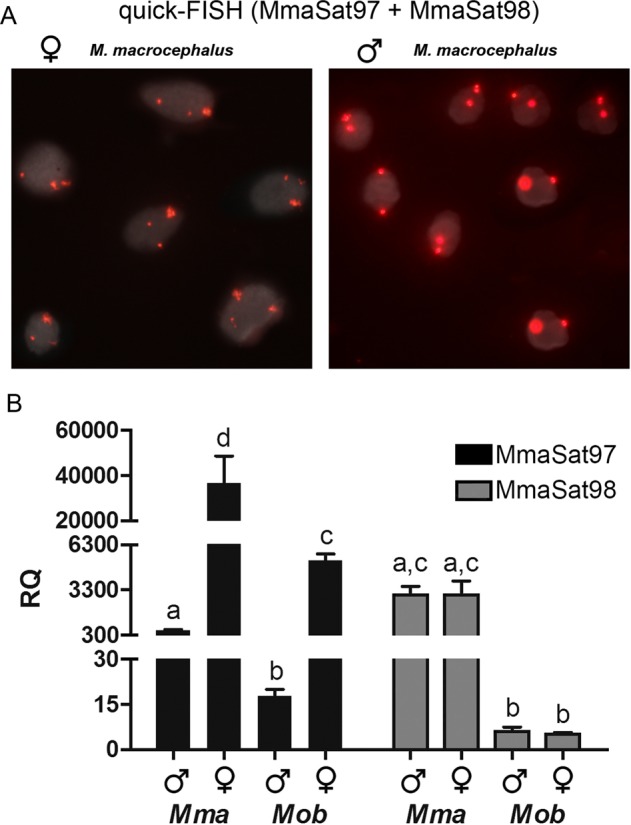
(**A**) quick-FISH results obtained in male and female samples. (**B**) Relative quantification (RQ) of MmaSat97 and MmaSat98 in males and females of *M*. *macrocephalus* and *M*. *obtusidens*. The letters indicate significant differences (P < 0.05) between samples.

For the quick-FISH approach, we selected MmaSat97, a W-specific satellite DNA, and MmaSat98, a satDNA located on a single pair of autosomes. Hybridization using digoxigenin-11-dUTP-labeled versions of these probes generates two signals in male cells, which correspond to the MmaSat98 *loci* within an autosomal pair, and three signals in female cells, which correspond to the two MmaSat98 loci and the additional MmaSat97 locus on the W chromosome. This approach was successfully applied to the identification of the sex of 20 live specimens. Cells derived from a single scale or a fin clip were hybridized with the two probes, and the results were satisfactory for both samples (Fig. 4). Different hybridization times were also tested (15 min, 1 h, 2 h and overnight), but no visible differences in terms of signal intensity were noticed (data not shown). As predicted, the female cells showed three conspicuous signals, while the male cells showed only two. Overall, the expected FISH signals were observed in almost 90% of all samples, which is likely due to the nuclear conformation within the slides and suggests that this method is 100% reliable for sex identification.

qPCR showed that the genomic abundance of MmaSat97 is different in males and females of *M*. *macrocephalus* and *M*. *obtusidens* (Fig. 4). This technique also revealed that the genomic abundance of MmaSat98 is similar in males and females of *M*. *macrocephalus* and of *M*. *obtusidens*, but is different in individuals of *M*. *macrocephalus* and *M*. *obtusidens* regardless of sex (Fig. 4). Overall, the sexing of individuals using the qPCR method we developed is also 100% reliable.

## Discussion

In the present study, we characterized 164 satDNA families in *M*. *macrocephalus* that were composed of 514 variants, which is the highest number of satellites characterized for a given species so far; of these, 64 families (155 variants) were grouped into 17 superfamilies. Considering that *Astyanax paranae* and *Characidium gomesi*, two other characiform species, contain 45 and 64 satDNA families, respectively^12^, it is likely that a great expansion of satellites occurred in the genome of *M*. *macrocephalus*.

It is well-known that satellites are highly dynamic sequences, which is directly related to the existence of several species- or group-specific satDNAs^2^. In this context, novel satellite DNA families may arise from the independent duplication of different genomic sequences, such as intergenic spacers, portions of transposable elements or even those derived from other satellite DNAs, which leads to a complex scenario^2,10^. Here, we found that almost half of the satDNAs in *M*. *macrocephalus* can be grouped into superfamilies, which demonstrates that satDNA expansion in this species is due to the duplication of existing repeats followed by substitutions/deletions/insertion events. Additionally, the existence of higher-order repeats (HORs), which contain alternate subrepeats with greater identity than contiguous repeats^4,42^, in some members of SF2 is evidence of the existence of another diversification mechanism within this species.

Remarkably, some of the selected satellites used for FISH belong to same superfamily, revealing that some satellites were subject to local duplication and amplification, such as those from SF6 (MmaSat113 and MmaSat139), SF15 (MmaSat158 and MmaSat62) and SF17 (MmaSat58 and MmaSat155) (Fig. 2 and Supplementary Fig. S1), while others were duplicated and disseminated to other chromosomal regions, such as SF10 (MmaSat61, 150 and 151) (Supplementary Fig. S1). Interestingly, one satDNA sequence with a length of 52 bp (MmaSat85-52) was found within the *A*. *paranae* (ApaSat29–52) and *C*. *gomesi* (CgomSat02–52) genomes^12^ (Serrano, unpublished), each of which exhibited different relative abundancies (with a few divergent positions; Supplementary Fig. S7). Considering the proposed phylogeny of the Characiformes order^43,44^, one must note that the referenced species (*M*. *macrocephalus*, *A*. *paranae* and *C*. *gomesi*) are distantly related characiform species, each belonging to a different family (Anostomidae, Characidae and Crenuchidae, respectively), which reveals the existence of an interesting and unusual case of satDNA conservation across an entire order, similar to that of the human alpha satellite^42^. Although we were not able to map this satDNA to particular chromosomes, the nucleotide conservation among the distantly related species (92.3% of mean sequence identity) is noteworthy, and further studies will be required to understand the dynamics of this repeat.

Our selection of satDNAs via the calculation of relative abundance values (female/male) was effective and revealed the presence of at least 22 satDNAs that had differentially accumulated within the heteromorphic sex chromosomes of *M*. *macrocephalus*, which suggested the presence of a high degree of differentiation between the Z and W chromosomes that was likely the result of a loss of recombination between these chromosomes, as has been theoretically predicted^13^. It is noteworthy that 18 out of the 22 satDNAs (78%) were retrieved exclusively during subsequent iterations of RepeatExplorer and DeconSeq that utilized the satMiner protocol^10^, which allows for the identification of low abundance satellites. These results will certainly produce an improved assembly for the W chromosome of this species by utilizing long reads. Most importantly, the existence of satDNAs that are exclusively clustered within the W chromosome (nonclustered in males) has provided an opportunity to corroborate the idea that low copy number satDNAs can escape from homogenization mechanisms^7,11,45^, as the divergence values of these specific satDNAs are higher in males.

As expected in a monophyletic ZZ/ZW system, our results demonstrated that some satellites are conserved in the W chromosomes of *M*. *macrocephalus* and *M*. *obtusidens*, which suggests that they were present in these chromosomes prior to the split of these species. On the other hand, there are satellites with differential accumulation in or that are exclusive to the W chromosome of *M*. *macrocephalus*, which corroborates the occurrence of an independent and continuous differentiation of the W chromosomes in this genus and is reinforced by the presence of some common satDNAs at different abundances^46^.

**Figure 5:**
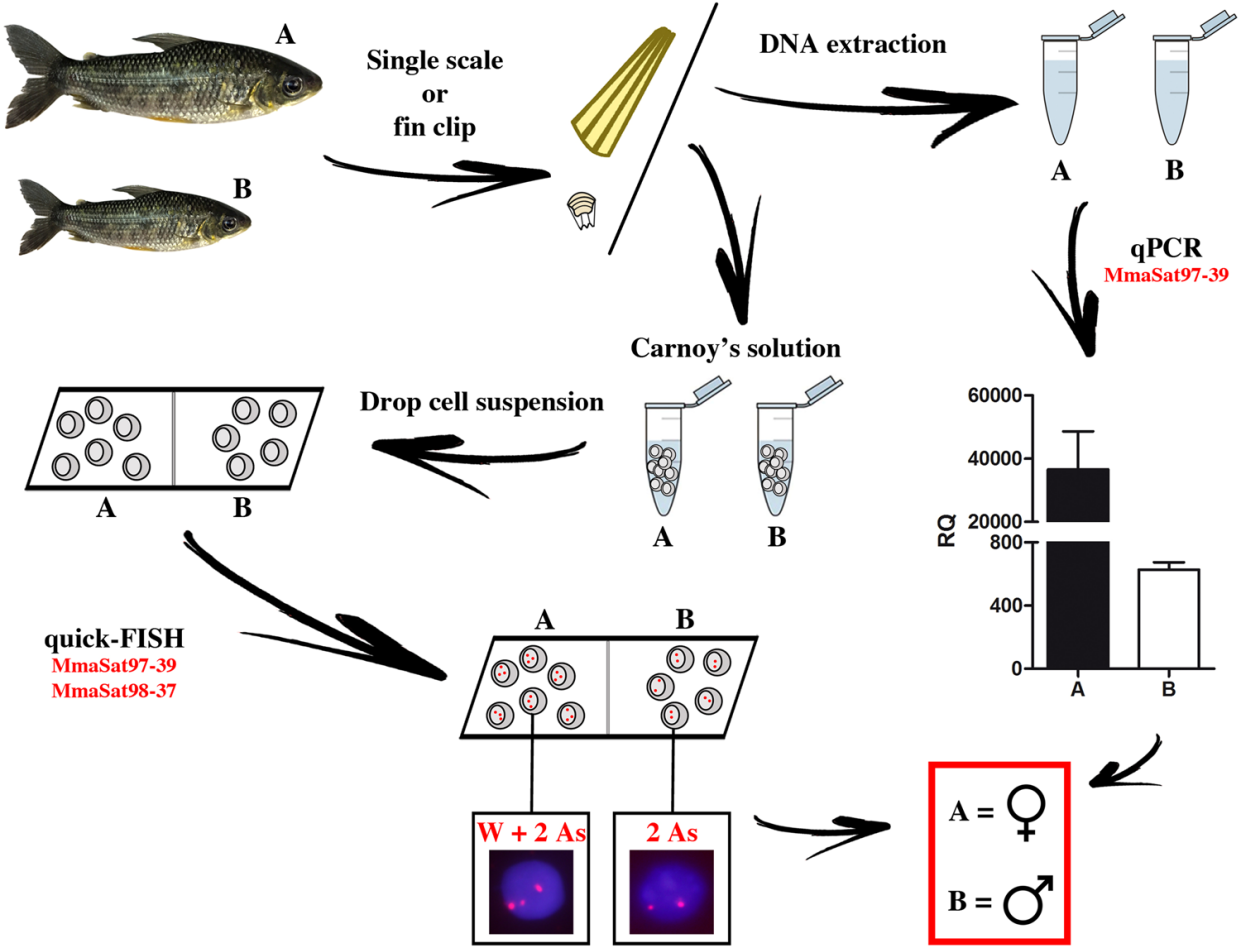
A schematic summary of the practical steps of utilizing satDNAs for sex identification in *M*. *macrocephalus*. From a single scale or a fin clip, it was possible to obtain cell suspensions or extract DNA and perform quick-FISH or qPCR, respectively.

From a practical perspective, the identified satDNAs that are differentially clustered on the W chromosomes of both species were valuable during the development of quick and noninvasive tools for sex identification in *M*. *macrocephalus* and *M*. *obtusidens*. Even though a conventional PCR-based approach for sex identification would be more practical for fish farmers^47^, one must note that both of the methods presented here are suitable for the identification of *Megaleporinus* superfemales (WW), which would be useful for aquaculture and future genomic studies (Fig. 5).

In summary, our study revealed the existence of a second fish species that has a characterized satellitome and allowed for the confirmation of some insights of satellite biology, the evolution of sex chromosomes and applications of satellite discoveries. The presented data, when combined with that from future analyses, will be useful for the precise characterization of the composition of the sex chromosomes in *Megaleporinus*. In addition, the development of quick and noninvasive tools for sexing individuals will be useful for future genomic and aquaculture studies.

## Supplementary information

Supplementary data 1
