# Supplementary material for: Satellitome landscape analysis of Megaleporinus macrocephalus (Teleostei, Anostomidae) reveals intense accumulation of satellite sequences on the heteromorphic sex chromosome
Source: Sci Rep. 2019 Apr 10;9:5856. doi: 10.1038/s41598-019-42383-8 (PMC6458115; doi:10.1038/s41598-019-42383-8)
Supplement: Supplementary file 1 — Supplementary data 1 [file 41598_2019_42383_MOESM1_ESM.pdf]

Supplementary information

Satellitome landscape analysis of *Megaleporinus macrocephalus* (Teleostei, Anostomidae) reveals intense accumulation of satellite sequences on the heteromorphic sex chromosome

Ricardo Utsunomia[1], Duílio Mazzoni Zerbinato de Andrade Silva[1], Francisco J. Ruiz-Ruano[2], Caio Augusto Gomes Goes[3], Silvana Melo[1], Lucas Peres Ramos[1], Claudio Oliveira[1], Fábio Porto Foresti[3], Fausto Foresti[1], Diogo Teruo Hashimoto[4]

[1] Departamento de Morfologia, Instituto de Biociências, Universidade Estadual Paulista - UNESP, Distrito de Rubião Junior, s/n, 18618-970, Botucatu, SP, Brazil. [2] Departamento de Genética, Universidad de Granada, 18071, Granada, Spain. [3]Departamento de Ciências Biológicas, Faculdade de Ciências, Universidade Estadual Paulista - UNESP, Campus de Bauru, 17033-360, Bauru, SP, Brazil. [4]CAUNESP, Universidade Estadual Paulista - UNESP, Campus Jaboticabal, 14884-900, Jaboticabal, SP, Brazil. Correspondence and requests for materials should be addressed to R.U. (email: ricardo.utsunomia@unesp.br)

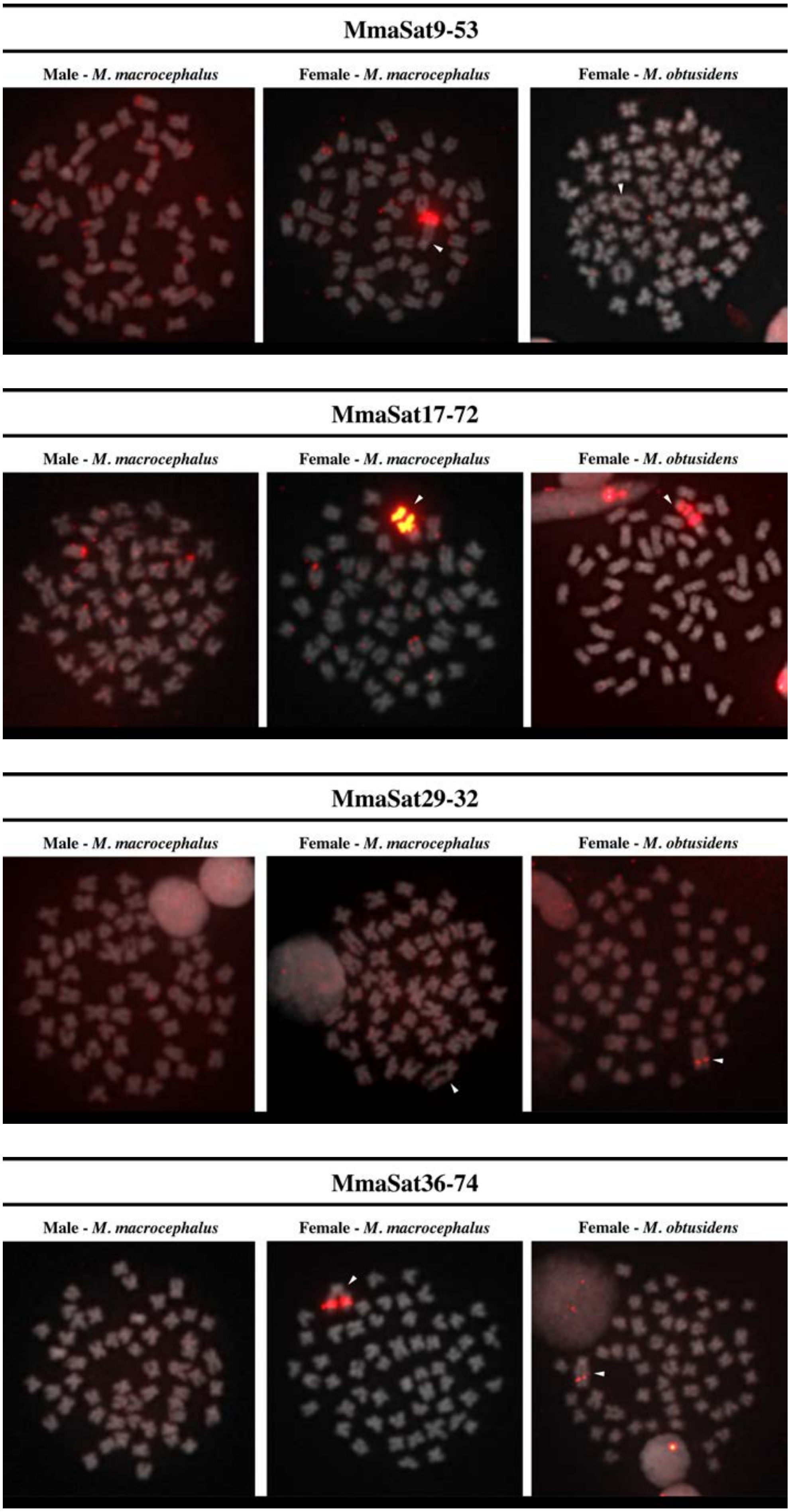
MmaSat9-53
Male - M. macrocephalus
Female - M. macrocephalus
Female - M. obtusidens
MmaSat17-72
Male - M. macrocephalus
Female - M. macrocephalus
Female - M. obtusidens
MmaSat29-32
Male - M. macrocephalus
Female - M. macrocephalus
Female - M. obtusidens
MmaSat36-74
Male - M. macrocephalus
Female - M. macrocephalus
Female - M. obtusidens

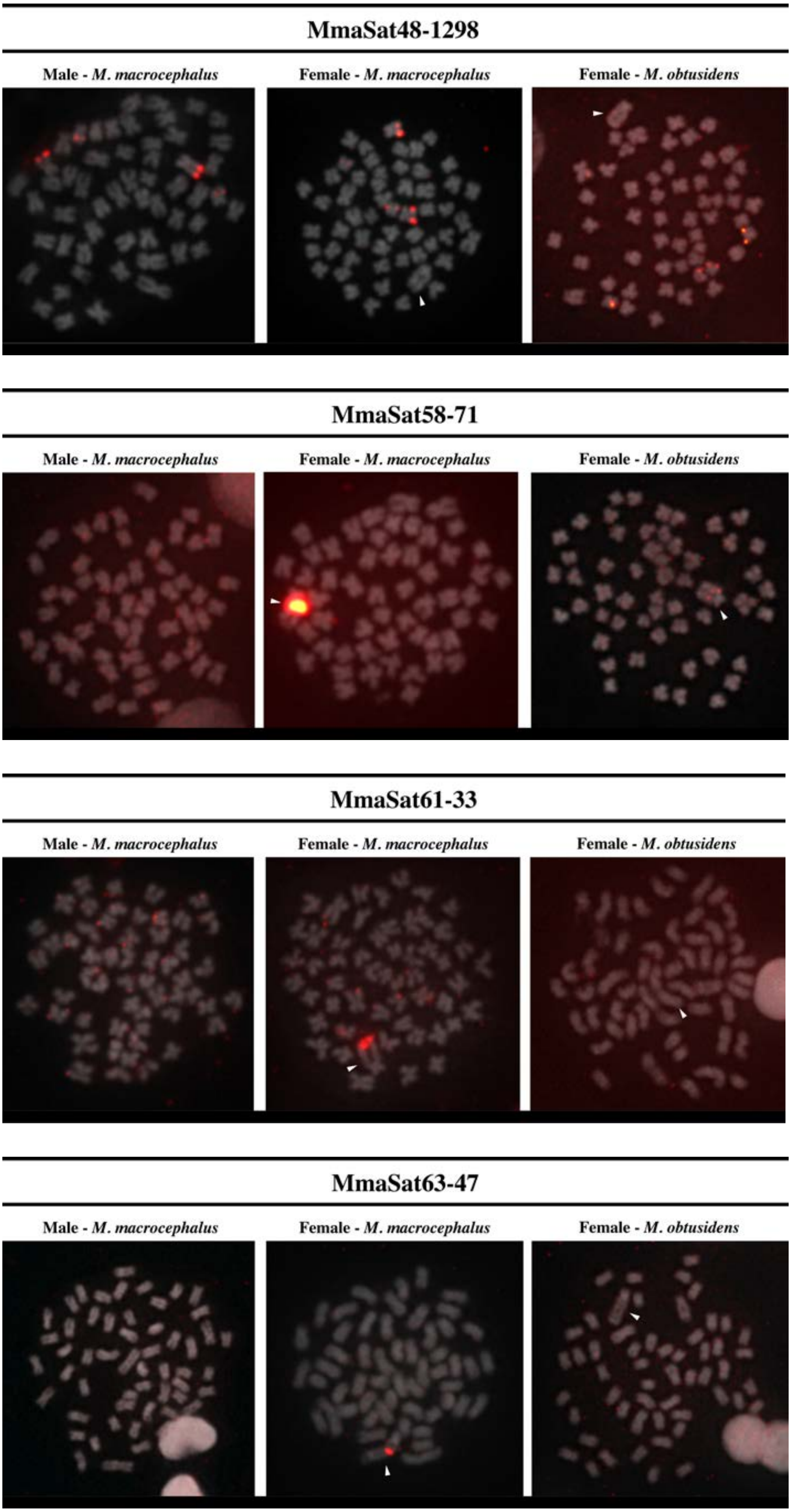
MmaSat48-1298
Male - M. macrocephalus
Female - M. macrocephalus
Female - M. obtusidens
MmaSat58-71
Male - M. macrocephalus
Female - M. macrocephalus
Female - M. obtusidens
MmaSat61-33
Male - M. macrocephalus
Female - M. macrocephalus
Female - M. obtusidens
MmaSat63-47
Male - M. macrocephalus
Female - M. macrocephalus
Female - M. obtusidens

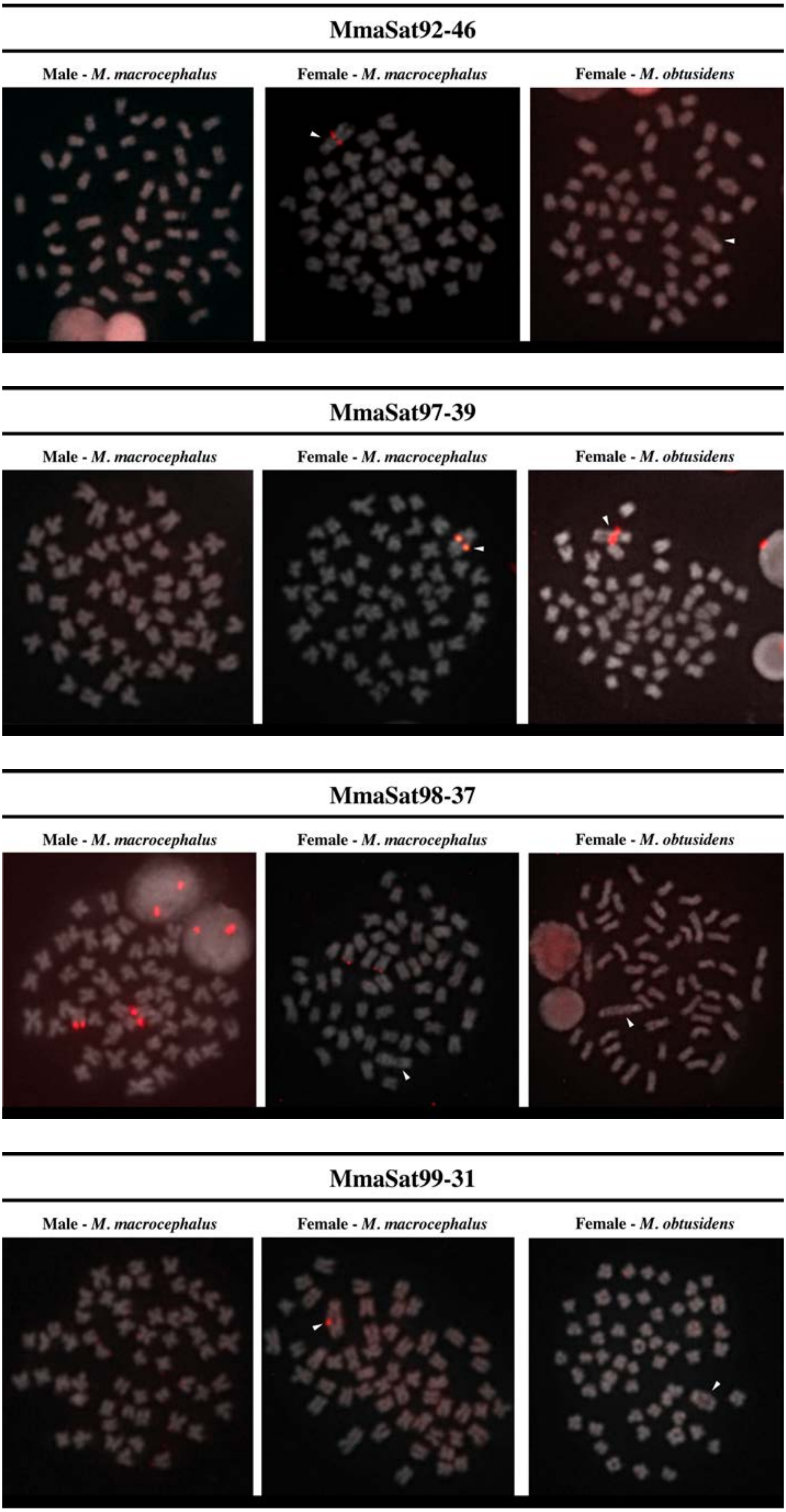
MmaSat92-46
Male - M. macrocephalus
Female - M. macrocephalus
Female - M. obtusidens
MmaSat97-39
Male - M. macrocephalus
Female - M. macrocephalus
Female - M. obtusidens
MmaSat98-37
Male - M. macrocephalus
Female - M. macrocephalus
Female - M. obtusidens
MmaSat99-31
Male - M. macrocephalus
Female - M. macrocephalus
Female - M. obtusidens

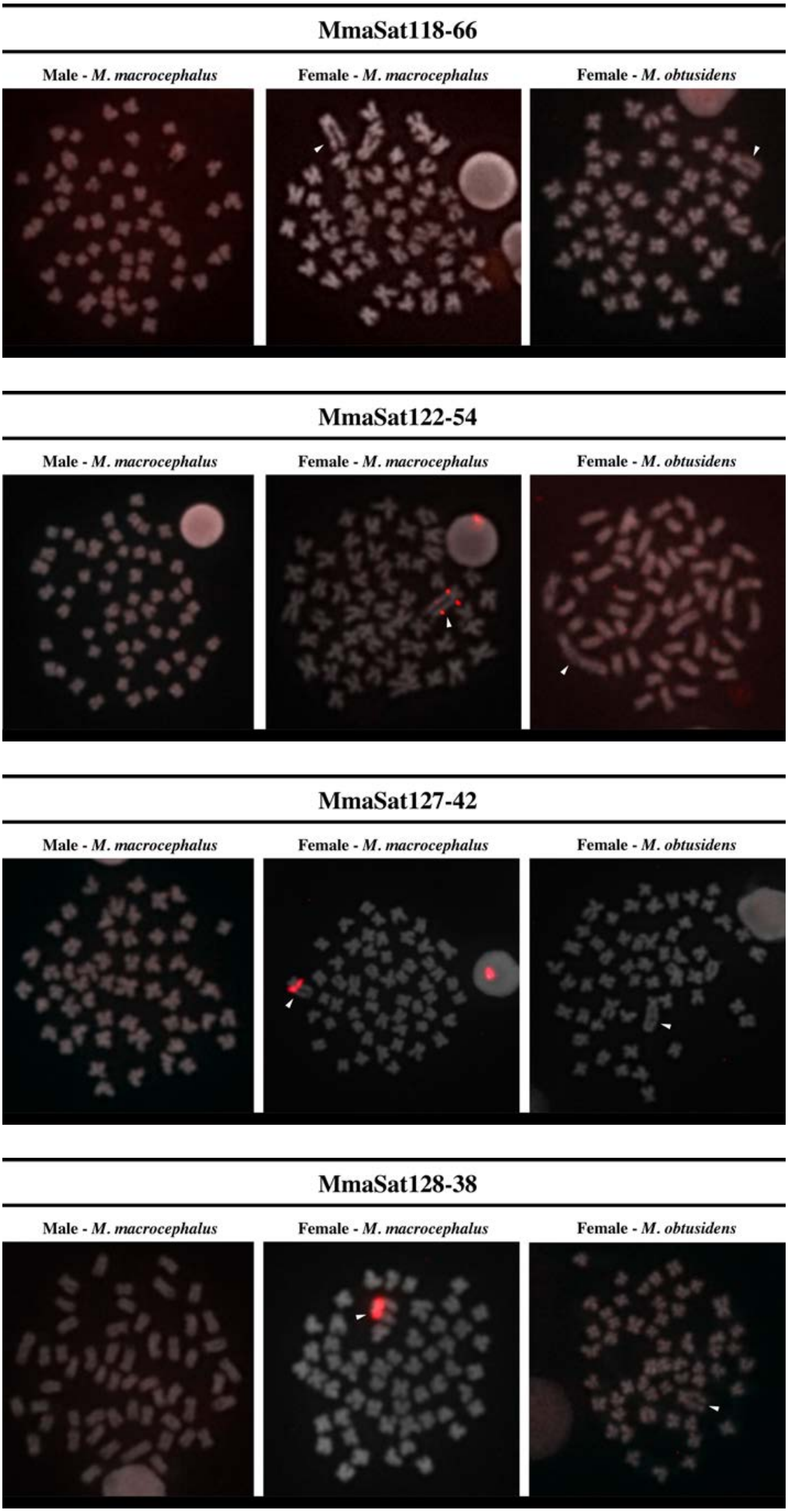
MmaSat118-66
Male - M. macrocephalus
Female - M. macrocephalus
Female - M. obtusidens
MmaSat122-54
Male - M. macrocephalus
Female - M. macrocephalus
Female - M. obtusidens
MmaSat127-42
Male - M. macrocephalus
Female - M. macrocephalus
Female - M. obtusidens
MmaSat128-38
Male - M. macrocephalus
Female - M. macrocephalus
Female - M. obtusidens

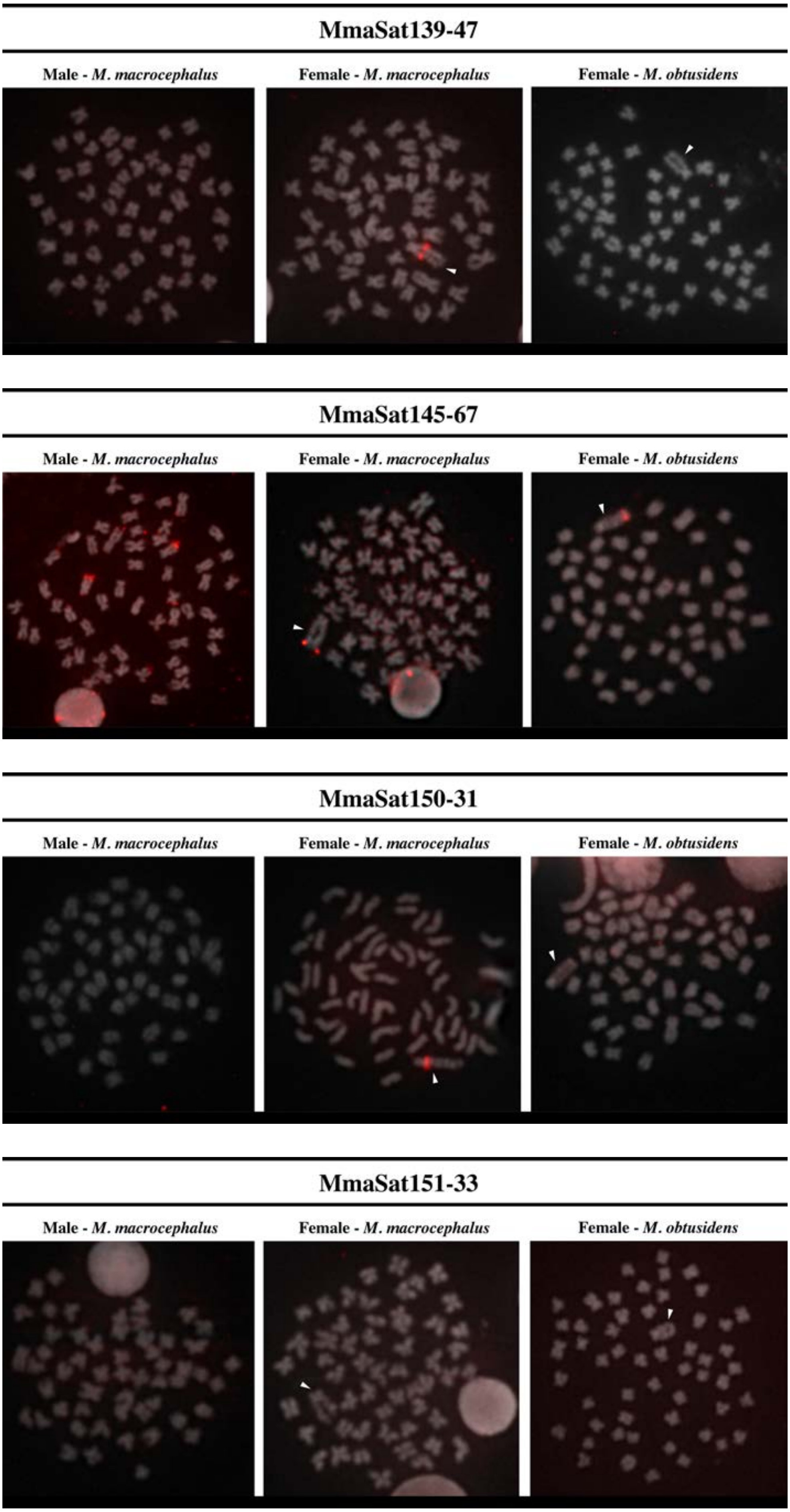
MmaSat139-47
Male - M. macrocephalus
Female - M. macrocephalus
Female - M. obtusidens
MmaSat145-67
Male - M. macrocephalus
Female - M. macrocephalus
Female - M. obtusidens
MmaSat150-31
Male - M. macrocephalus
Female - M. macrocephalus
Female - M. obtusidens
MmaSat151-33
Male - M. macrocephalus
Female - M. macrocephalus
Female - M. obtusidens

MmaSat152-31

Male - *M. macrocephalus* | **Female - *M. macrocephalus*** | **Female - *M. obtusidens***

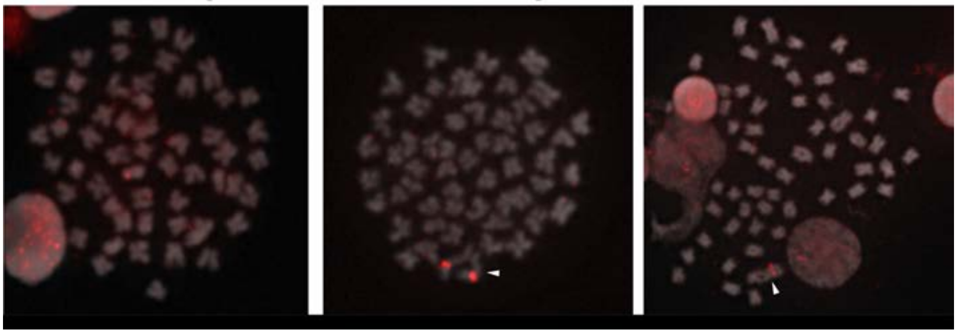

MmaSat153-40

Male - *M. macrocephalus* | **Female - *M. macrocephalus*** | **Female - *M. obtusidens***

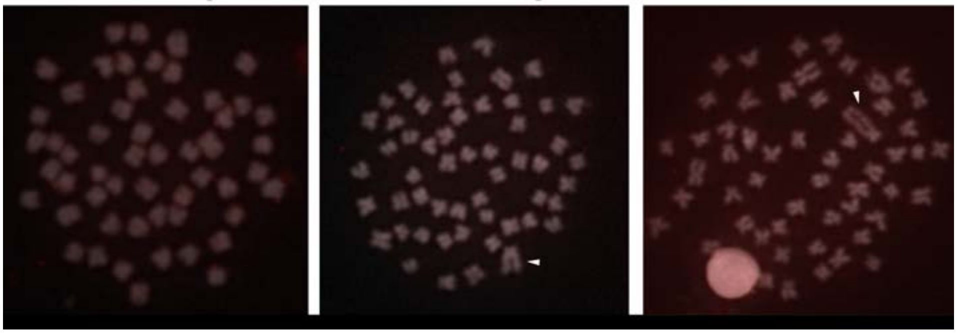

MmaSat154-30

Male - *M. macrocephalus* | **Female - *M. macrocephalus*** | **Female - *M. obtusidens***

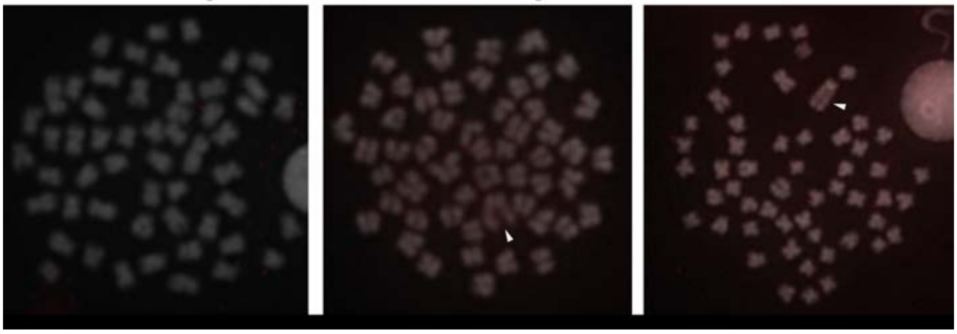

MmaSat155-71

Male - *M. macrocephalus* | **Female - *M. macrocephalus*** | **Female - *M. obtusidens***

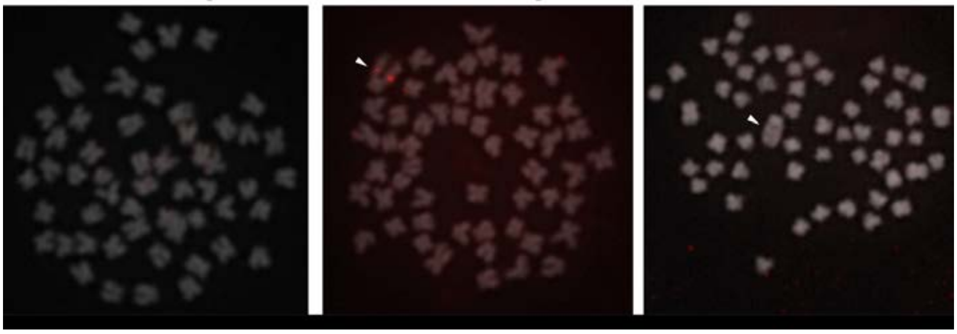

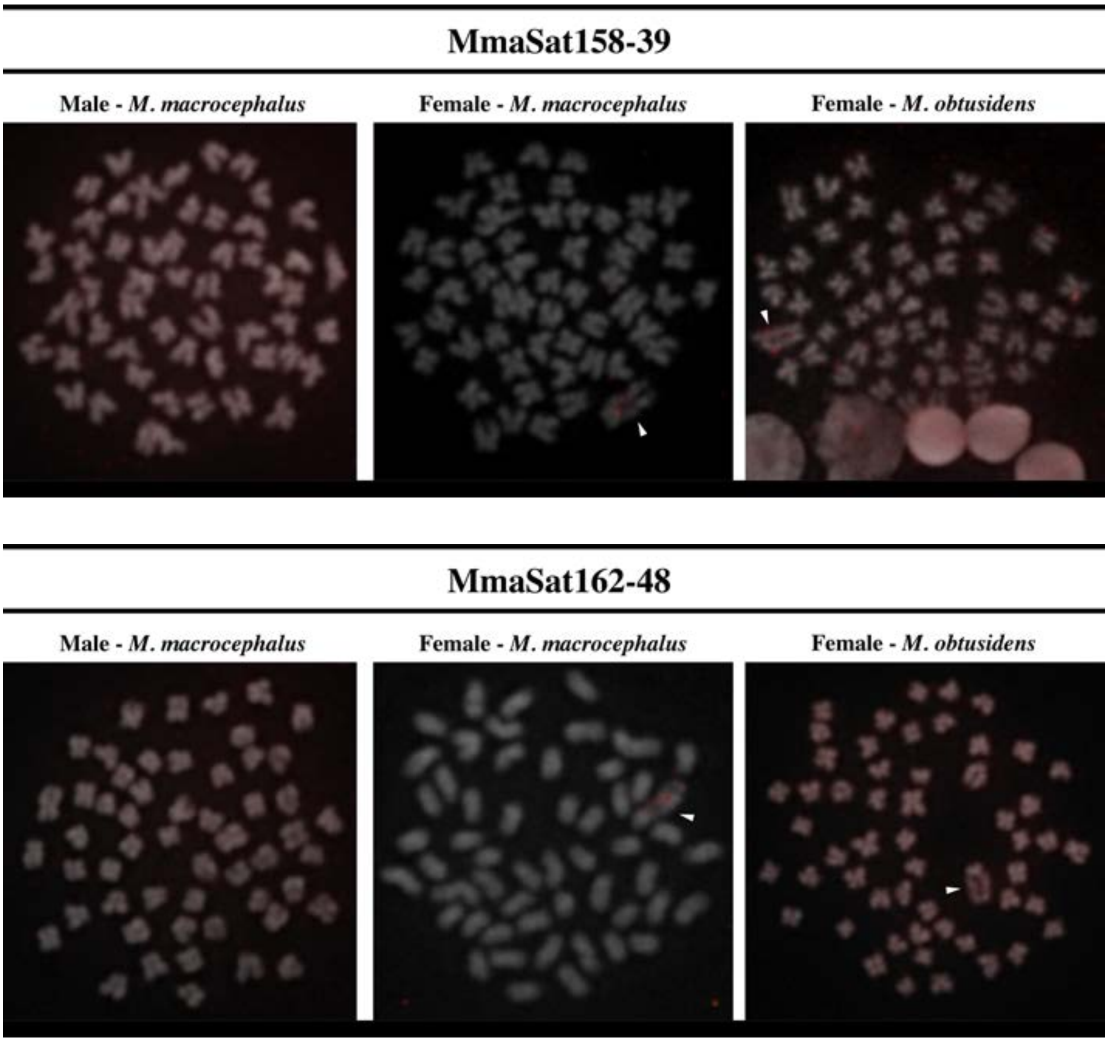

Supplementary Figure S1: Metaphase plates from *M*. *macrocephalus* (males and females) and *M*. *obtusidens* (females) after FISH identification of the selected satDNA families.

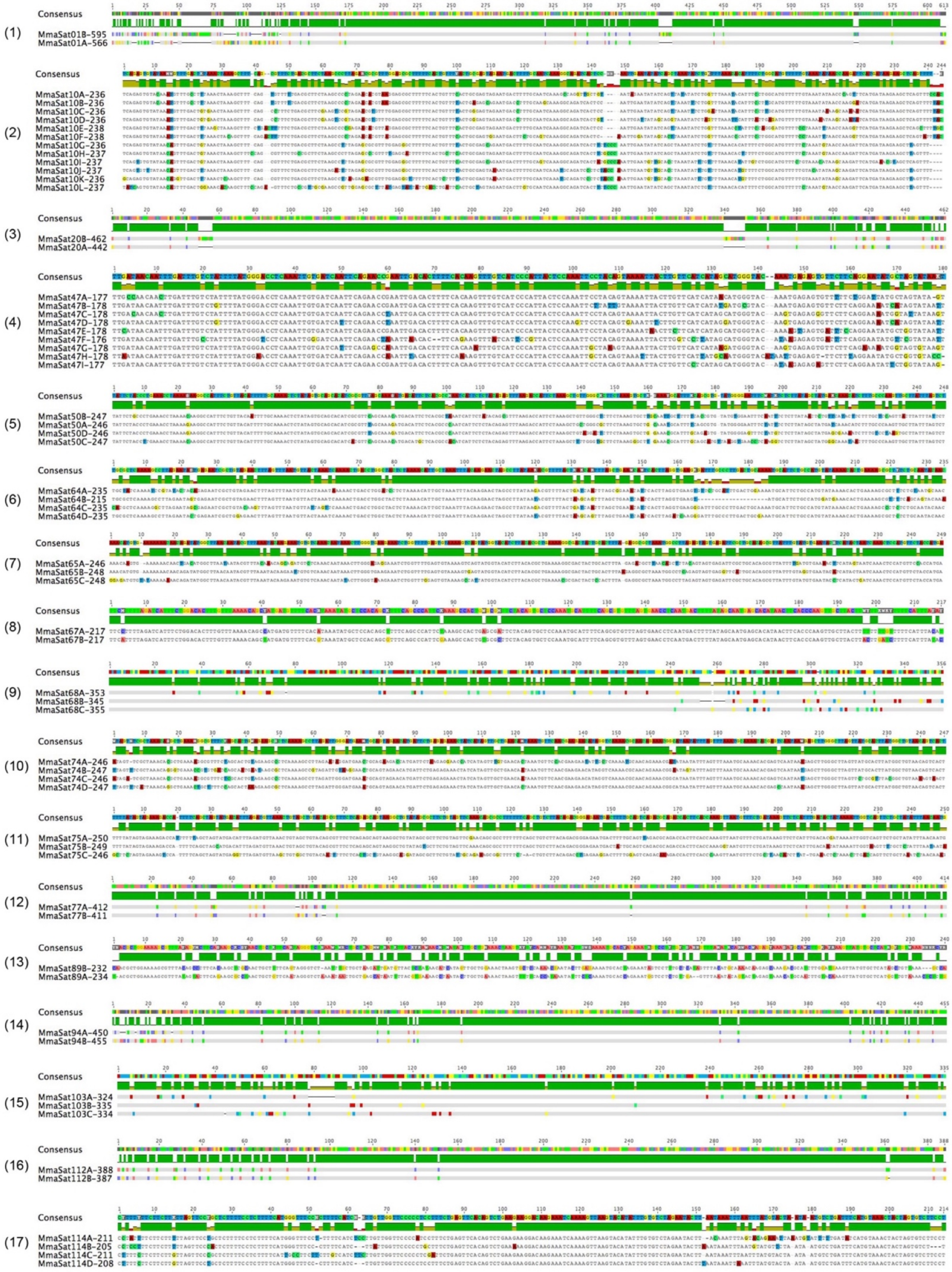

(1)
Consensus
MmaSat01B-595
MmaSat01A-566
(2)
Consensus
MmaSat10A-236
MmaSat10B-236
MmaSat10C-236
MmaSat10D-236
MmaSat10E-238
MmaSat10F-238
MmaSat10G-236
MmaSat10H-237
MmaSat10I-237
MmaSat10J-237
MmaSat10K-236
MmaSat10L-237
(3)
Consensus
MmaSat20B-462
MmaSat20A-442
(4)
Consensus
MmaSat47A-177
MmaSat47B-178
MmaSat47C-178
MmaSat47D-178
MmaSat47E-178
MmaSat47F-176
MmaSat47G-178
MmaSat47H-178
MmaSat47I-177
(5)
Consensus
MmaSat50B-247
MmaSat50A-246
MmaSat50D-246
MmaSat50C-247
(6)
Consensus
MmaSat64A-235
MmaSat64B-215
MmaSat64C-235
MmaSat64D-235
(7)
Consensus
MmaSat65A-246
MmaSat65B-248
MmaSat65C-248
(8)
Consensus
MmaSat67A-217
MmaSat67B-217
(9)
Consensus
MmaSat68A-353
MmaSat68B-345
MmaSat68C-355
(10)
Consensus
MmaSat74A-246
MmaSat74B-247
MmaSat74C-246
MmaSat74D-247
(11)
Consensus
MmaSat75A-250
MmaSat75B-249
MmaSat75C-246
(12)
Consensus
MmaSat77A-412
MmaSat77B-411
(13)
Consensus
MmaSat89B-232
MmaSat89A-234
(14)
Consensus
MmaSat94A-450
MmaSat94B-455
(15)
Consensus
MmaSat103A-324
MmaSat103B-335
MmaSat103C-334
(16)
Consensus
MmaSat112A-388
MmaSat112B-387
(17)
Consensus
MmaSat114A-211
MmaSat114B-205
MmaSat114C-211
MmaSat114D-208

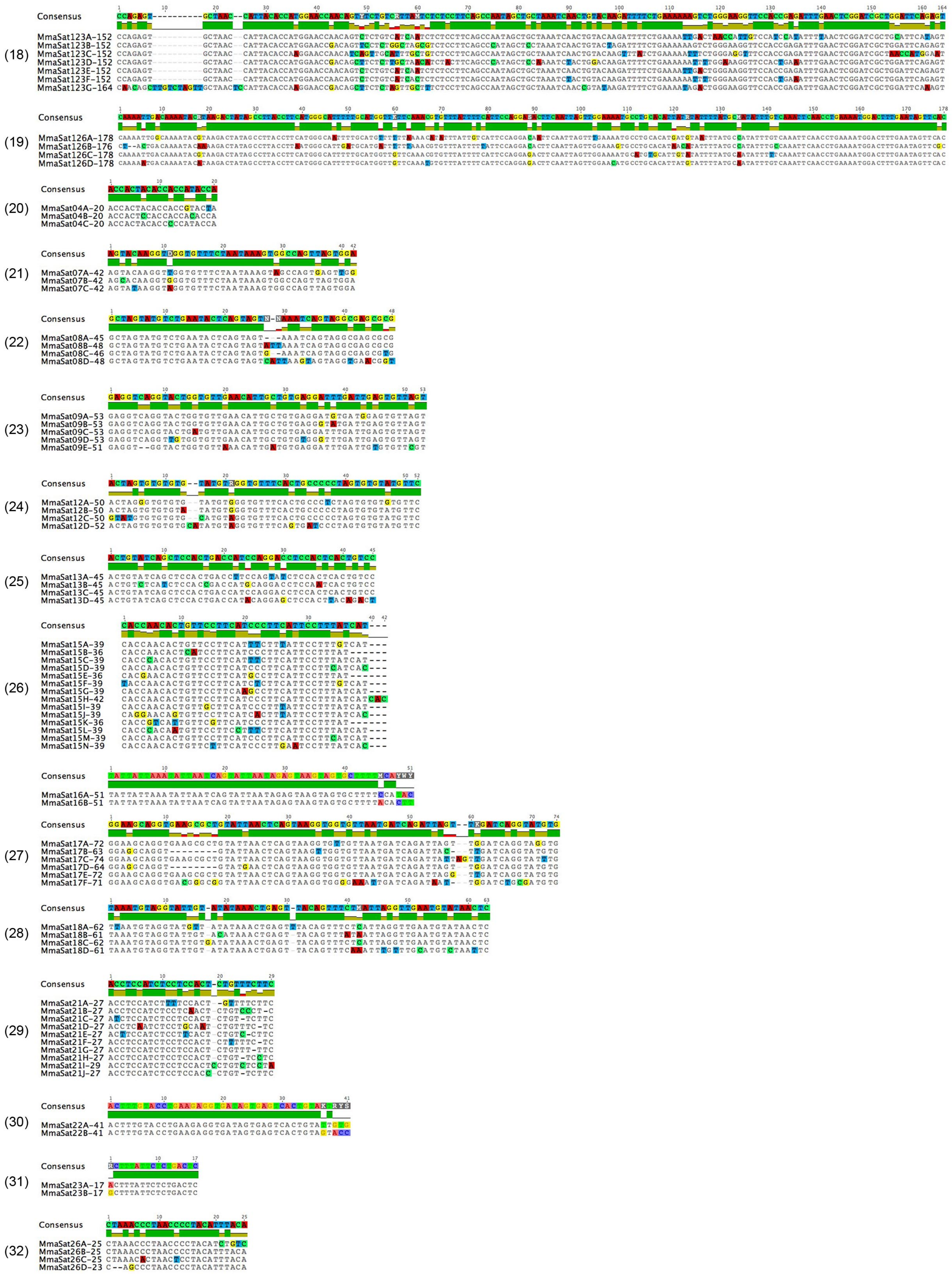

(18)
Consensus
MmaSat123A-152
MmaSat123B-152
MmaSat123C-152
MmaSat123D-152
MmaSat123E-152
MmaSat123F-152
MmaSat123G-164
(19)
Consensus
MmaSat126A-178
MmaSat126B-176
MmaSat126C-178
MmaSat126D-178
(20)
Consensus
MmaSat04A-20
MmaSat04B-20
MmaSat04C-20
(21)
Consensus
MmaSat07A-42
MmaSat07B-42
MmaSat07C-42
(22)
Consensus
MmaSat08A-45
MmaSat08B-48
MmaSat08C-46
MmaSat08D-48
(23)
Consensus
MmaSat09A-53
MmaSat09B-53
MmaSat09C-53
MmaSat09D-53
MmaSat09E-51
(24)
Consensus
MmaSat12A-50
MmaSat12B-50
MmaSat12C-50
MmaSat12D-52
(25)
Consensus
MmaSat13A-45
MmaSat13B-45
MmaSat13C-45
MmaSat13D-45
(26)
Consensus
MmaSat15A-39
MmaSat15B-36
MmaSat15C-39
MmaSat15D-39
MmaSat15E-36
MmaSat15F-39
MmaSat15G-39
MmaSat15H-42
MmaSat15I-39
MmaSat15J-39
MmaSat15K-36
MmaSat15L-39
MmaSat15M-39
MmaSat15N-39
(27)
Consensus
MmaSat16A-51
MmaSat16B-51
Consensus
MmaSat17A-72
MmaSat17B-63
MmaSat17C-74
MmaSat17D-64
MmaSat17E-72
MmaSat17F-71
(28)
Consensus
MmaSat18A-62
MmaSat18B-61
MmaSat18C-62
MmaSat18D-61
(29)
Consensus
MmaSat21A-27
MmaSat21B-27
MmaSat21C-27
MmaSat21D-27
MmaSat21E-27
MmaSat21F-27
MmaSat21G-27
MmaSat21H-27
MmaSat21I-29
MmaSat21J-27
(30)
Consensus
MmaSat22A-41
MmaSat22B-41
(31)
Consensus
MmaSat23A-17
MmaSat23B-17
(32)
Consensus
MmaSat26A-25
MmaSat26B-25
MmaSat26C-25
MmaSat26D-23

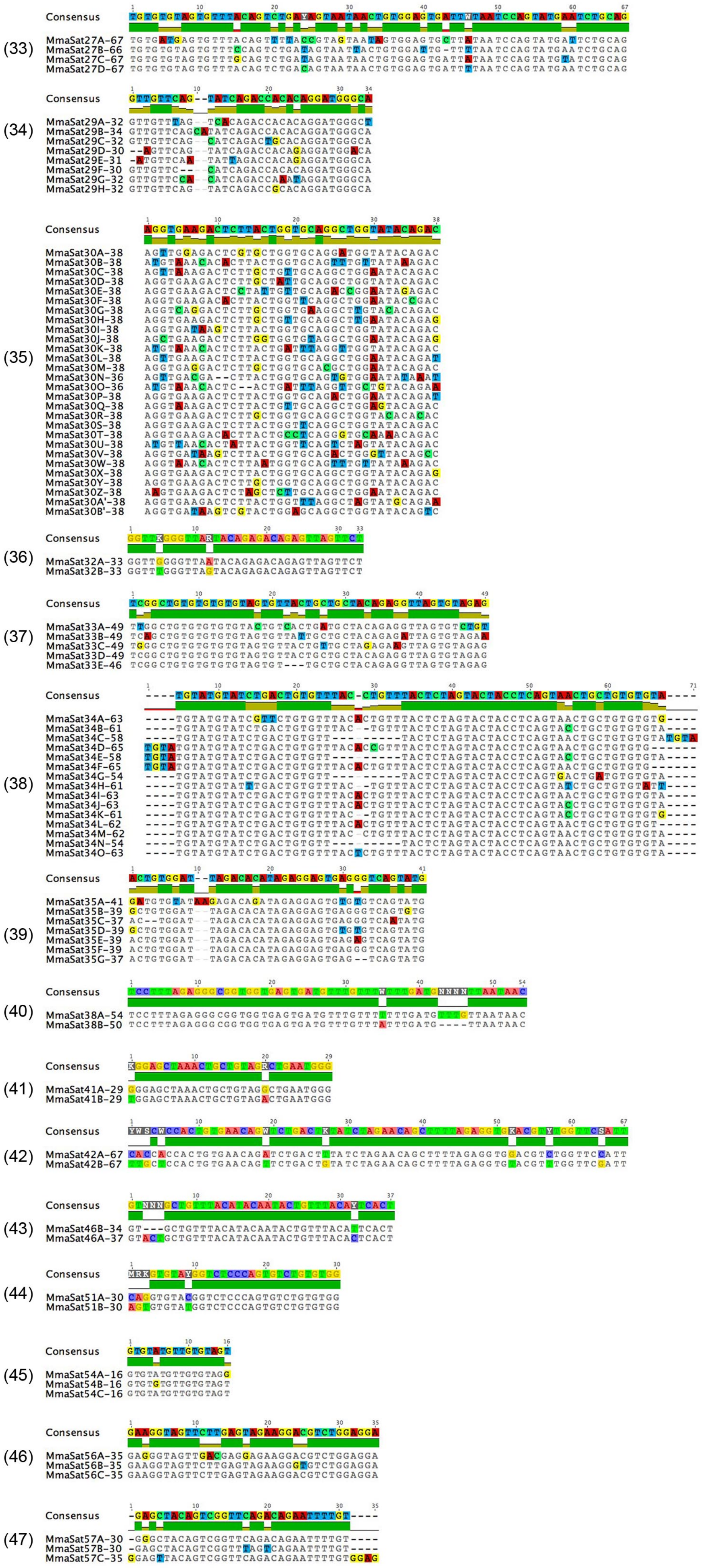
(33)
Consensus
MmaSat27A-67
MmaSat27B-66
MmaSat27C-67
MmaSat27D-67
(34)
Consensus
MmaSat29A-32
MmaSat29B-34
MmaSat29C-32
MmaSat29D-30
MmaSat29E-31
MmaSat29F-30
MmaSat29G-32
MmaSat29H-32
(35)
Consensus
MmaSat30A-38
MmaSat30B-38
MmaSat30C-38
MmaSat30D-38
MmaSat30E-38
MmaSat30F-38
MmaSat30G-38
MmaSat30H-38
MmaSat30I-38
MmaSat30J-38
MmaSat30K-38
MmaSat30L-38
MmaSat30M-38
MmaSat30N-36
MmaSat30O-36
MmaSat30P-38
MmaSat30Q-38
MmaSat30R-38
MmaSat30S-38
MmaSat30T-38
MmaSat30U-38
MmaSat30V-38
MmaSat30W-38
MmaSat30X-38
MmaSat30Y-38
MmaSat30Z-38
MmaSat30A'-38
MmaSat30B'-38
(36)
Consensus
MmaSat32A-33
MmaSat32B-33
(37)
Consensus
MmaSat33A-49
MmaSat33B-49
MmaSat33C-49
MmaSat33D-49
MmaSat33E-46
(38)
Consensus
MmaSat34A-63
MmaSat34B-61
MmaSat34C-58
MmaSat34D-65
MmaSat34E-58
MmaSat34F-65
MmaSat34G-54
MmaSat34H-61
MmaSat34I-63
MmaSat34J-63
MmaSat34K-61
MmaSat34L-62
MmaSat34M-62
MmaSat34N-54
MmaSat34O-63
(39)
Consensus
MmaSat35A-41
MmaSat35B-39
MmaSat35C-37
MmaSat35D-39
MmaSat35E-39
MmaSat35F-39
MmaSat35G-37
(40)
Consensus
MmaSat38A-54
MmaSat38B-50
(41)
Consensus
MmaSat41A-29
MmaSat41B-29
(42)
Consensus
MmaSat42A-67
MmaSat42B-67
(43)
Consensus
MmaSat46B-34
MmaSat46A-37
(44)
Consensus
MmaSat51A-30
MmaSat51B-30
(45)
Consensus
MmaSat54A-16
MmaSat54B-16
MmaSat54C-16
(46)
Consensus
MmaSat56A-35
MmaSat56B-35
MmaSat56C-35
(47)
Consensus
MmaSat57A-30
MmaSat57B-30
MmaSat57C-35

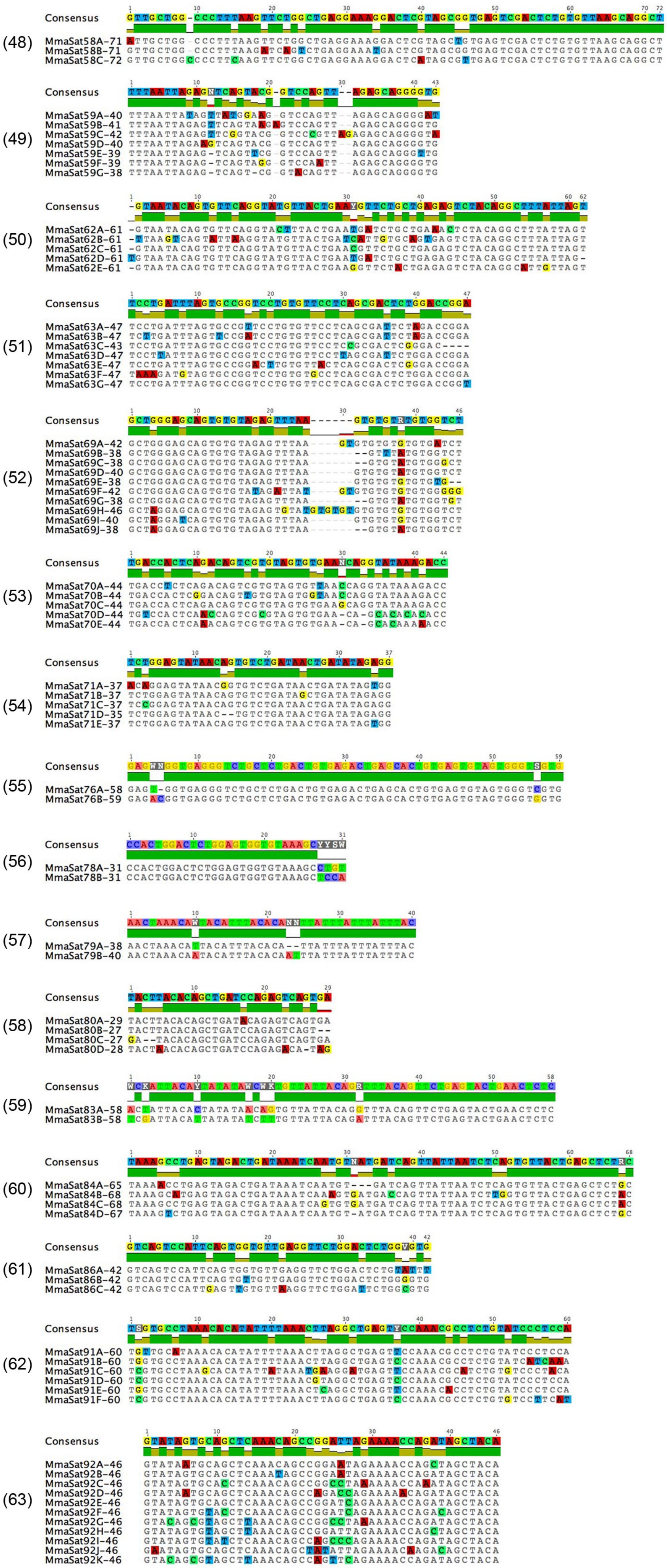

(48)
MmaSat58A-71
MmaSat58B-71
MmaSat58C-72
(49)
MmaSat59A-40
MmaSat59B-41
MmaSat59C-42
MmaSat59D-40
MmaSat59E-39
MmaSat59F-39
MmaSat59G-38
(50)
MmaSat62A-61
MmaSat62B-61
MmaSat62C-61
MmaSat62D-61
MmaSat62E-61
(51)
MmaSat63A-47
MmaSat63B-47
MmaSat63C-43
MmaSat63D-47
MmaSat63E-47
MmaSat63F-47
MmaSat63G-47
(52)
MmaSat69A-42
MmaSat69B-38
MmaSat69C-38
MmaSat69D-40
MmaSat69E-38
MmaSat69F-42
MmaSat69G-38
MmaSat69H-46
MmaSat69I-40
MmaSat69J-38
(53)
MmaSat70A-44
MmaSat70B-44
MmaSat70C-44
MmaSat70D-44
MmaSat70E-44
(54)
MmaSat71A-37
MmaSat71B-37
MmaSat71C-37
MmaSat71D-35
MmaSat71E-37
(55)
MmaSat76A-58
MmaSat76B-59
(56)
MmaSat78A-31
MmaSat78B-31
(57)
MmaSat79A-38
MmaSat79B-40
(58)
MmaSat80A-29
MmaSat80B-27
MmaSat80C-27
MmaSat80D-28
(59)
MmaSat83A-58
MmaSat83B-58
(60)
MmaSat84A-65
MmaSat84B-68
MmaSat84C-68
MmaSat84D-67
(61)
MmaSat86A-42
MmaSat86B-42
MmaSat86C-42
(62)
MmaSat91A-60
MmaSat91B-60
MmaSat91C-60
MmaSat91D-60
MmaSat91E-60
MmaSat91F-60
(63)
MmaSat92A-46
MmaSat92B-46
MmaSat92C-46
MmaSat92D-46
MmaSat92E-46
MmaSat92F-46
MmaSat92G-46
MmaSat92H-46
MmaSat92I-46
MmaSat92J-46
MmaSat92K-46

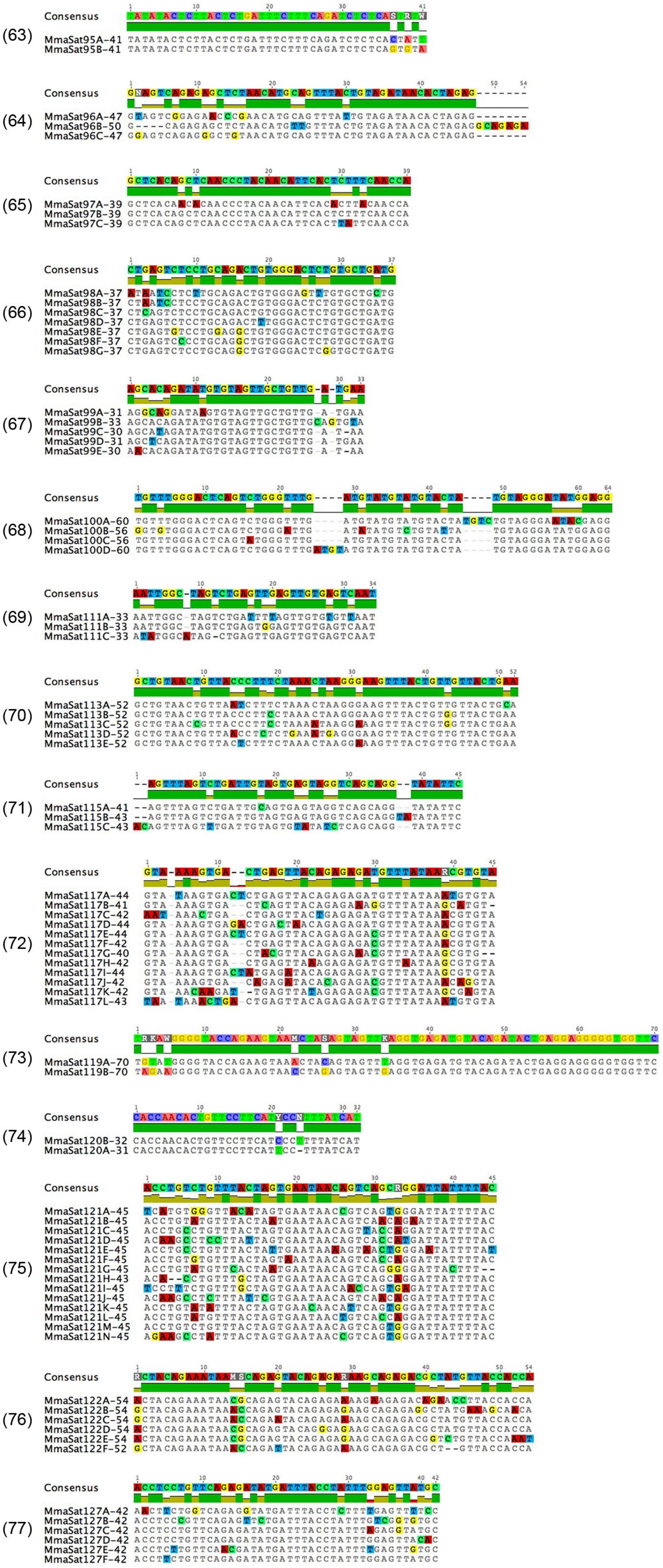
(63)
Consensus
MmaSat95A-41
MmaSat95B-41
(64)
Consensus
MmaSat96A-47
MmaSat96B-50
MmaSat96C-47
(65)
Consensus
MmaSat97A-39
MmaSat97B-39
MmaSat97C-39
(66)
Consensus
MmaSat98A-37
MmaSat98B-37
MmaSat98C-37
MmaSat98D-37
MmaSat98E-37
MmaSat98F-37
MmaSat98G-37
(67)
Consensus
MmaSat99A-31
MmaSat99B-33
MmaSat99C-30
MmaSat99D-31
MmaSat99E-30
(68)
Consensus
MmaSat100A-60
MmaSat100B-56
MmaSat100C-56
MmaSat100D-60
(69)
Consensus
MmaSat111A-33
MmaSat111B-33
MmaSat111C-33
(70)
Consensus
MmaSat113A-52
MmaSat113B-52
MmaSat113C-52
MmaSat113D-52
MmaSat113E-52
(71)
Consensus
MmaSat115A-41
MmaSat115B-43
MmaSat115C-43
(72)
Consensus
MmaSat117A-44
MmaSat117B-41
MmaSat117C-42
MmaSat117D-44
MmaSat117E-44
MmaSat117F-42
MmaSat117G-40
MmaSat117H-42
MmaSat117I-44
MmaSat117J-42
MmaSat117K-42
MmaSat117L-43
(73)
Consensus
MmaSat119A-70
MmaSat119B-70
(74)
Consensus
MmaSat120B-32
MmaSat120A-31
(75)
Consensus
MmaSat121A-45
MmaSat121B-45
MmaSat121C-45
MmaSat121D-45
MmaSat121E-45
MmaSat121F-45
MmaSat121G-45
MmaSat121H-43
MmaSat121I-45
MmaSat121J-45
MmaSat121K-45
MmaSat121L-45
MmaSat121M-45
MmaSat121N-45
(76)
Consensus
MmaSat122A-54
MmaSat122B-54
MmaSat122C-54
MmaSat122D-54
MmaSat122E-54
MmaSat122F-52
(77)
Consensus
MmaSat127A-42
MmaSat127B-42
MmaSat127C-42
MmaSat127D-42
MmaSat127E-42
MmaSat127F-42

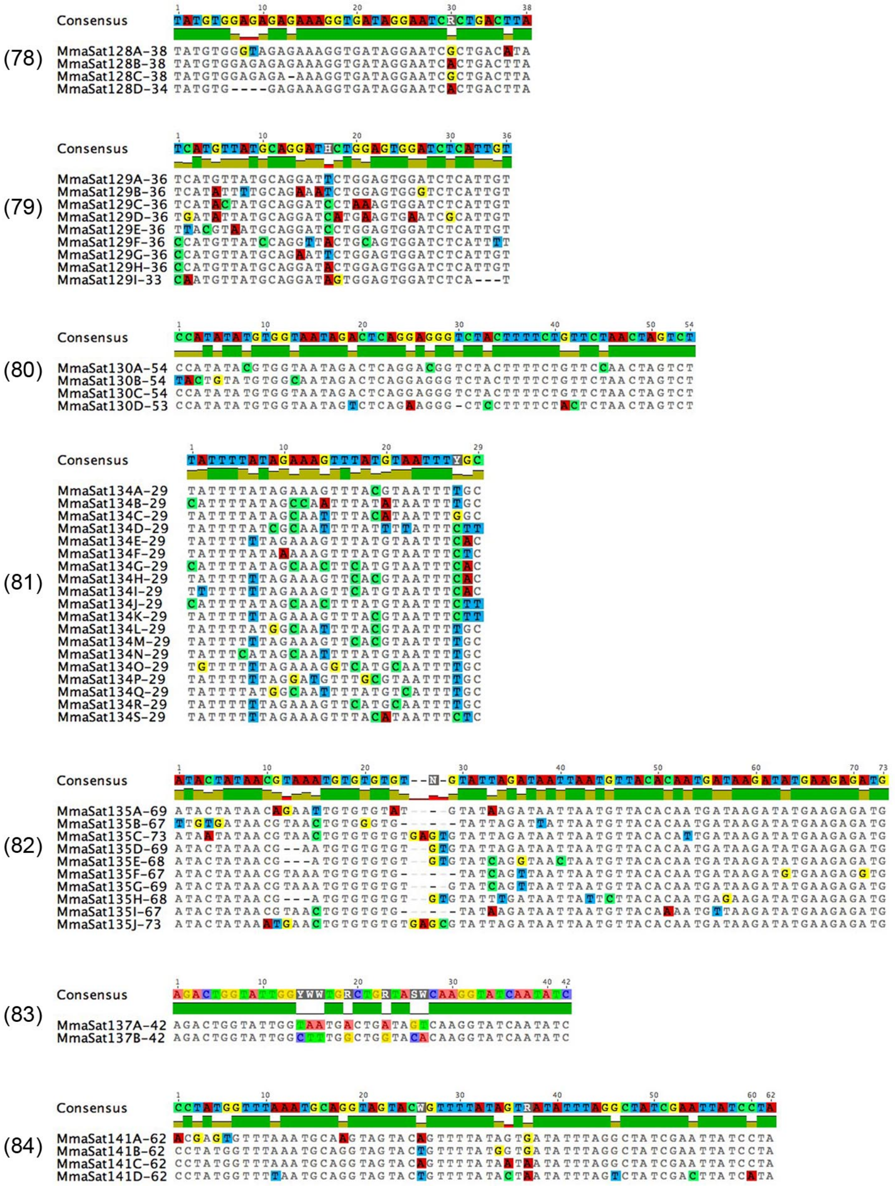

Supplementary Figure S2: Alignments of the different variants that represented several long (1-19) and short (20-84) satDNA families.

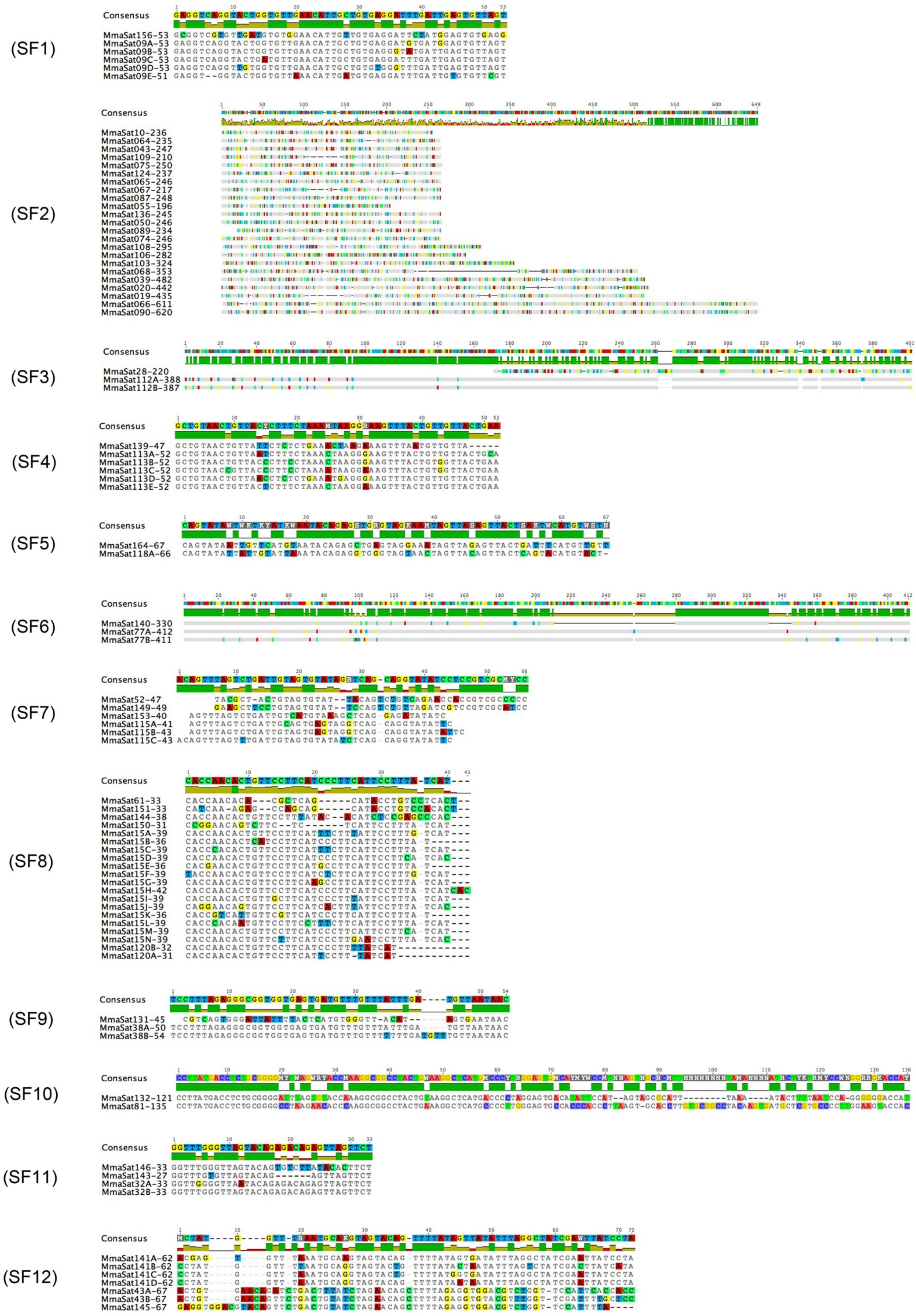
(SF1)
Consensus
MmaSat156-53
MmaSat09A-53
MmaSat09B-53
MmaSat09C-53
MmaSat09D-53
MmaSat09E-51
(SF2)
Consensus
MmaSat10-236
MmaSat064-235
MmaSat043-247
MmaSat109-210
MmaSat075-250
MmaSat124-237
MmaSat065-246
MmaSat067-217
MmaSat087-248
MmaSat055-196
MmaSat136-245
MmaSat050-246
MmaSat089-234
MmaSat074-246
MmaSat108-295
MmaSat106-282
MmaSat103-324
MmaSat068-353
MmaSat039-482
MmaSat020-442
MmaSat019-435
MmaSat066-611
MmaSat090-620
(SF3)
Consensus
MmaSat28-220
MmaSat112A-388
MmaSat112B-387
(SF4)
Consensus
MmaSat139-47
MmaSat113A-52
MmaSat113B-52
MmaSat113C-52
MmaSat113D-52
MmaSat113E-52
(SF5)
Consensus
MmaSat164-67
MmaSat118A-66
(SF6)
Consensus
MmaSat140-330
MmaSat77A-412
MmaSat77B-411
(SF7)
Consensus
MmaSat52-47
MmaSat149-49
MmaSat153-40
MmaSat115A-41
MmaSat115B-43
MmaSat115C-43
(SF8)
Consensus
MmaSat61-33
MmaSat151-33
MmaSat144-38
MmaSat150-31
MmaSat15A-39
MmaSat15B-36
MmaSat15C-39
MmaSat15D-39
MmaSat15E-36
MmaSat15F-39
MmaSat15G-39
MmaSat15H-42
MmaSat15I-39
MmaSat15J-39
MmaSat15K-36
MmaSat15L-39
MmaSat15M-39
MmaSat15N-39
MmaSat120B-32
MmaSat120A-31
(SF9)
Consensus
MmaSat131-45
MmaSat38A-50
MmaSat38B-54
(SF10)
Consensus
MmaSat132-121
MmaSat81-135
(SF11)
Consensus
MmaSat146-33
MmaSat143-27
MmaSat32A-33
MmaSat32B-33
(SF12)
Consensus
MmaSat141A-62
MmaSat141B-62
MmaSat141C-62
MmaSat141D-62
MmaSat43A-67
MmaSat43B-67
MmaSat145-67

(SF13)

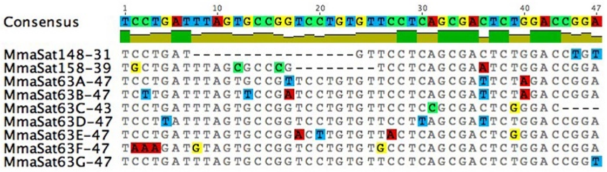

(SF14)

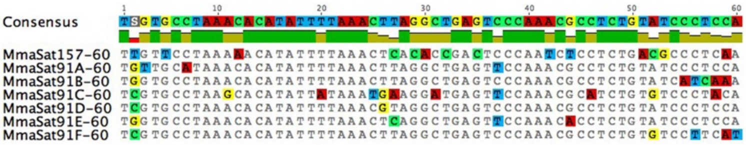

(SF15)

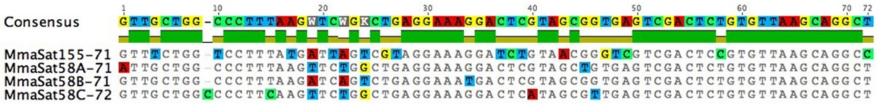

(SF16)

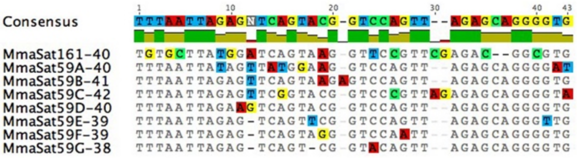

(SF17)

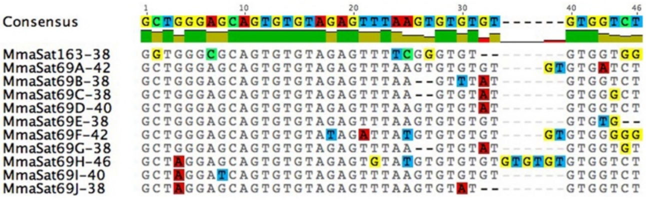

Conserved motifs between long satDNAs

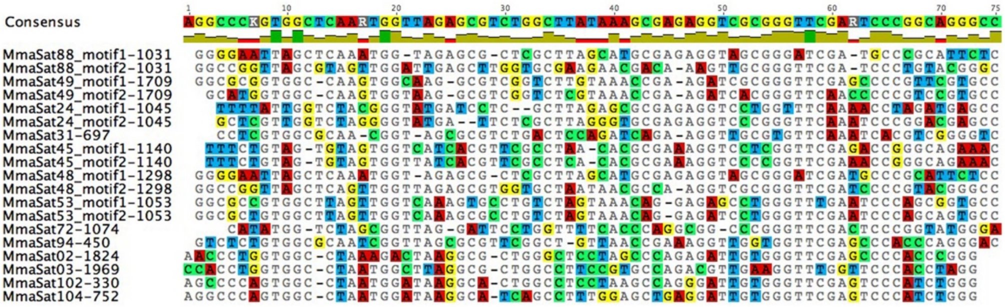

Supplementary Figure S3: Alignments between sequences from the characterized superfamilies.

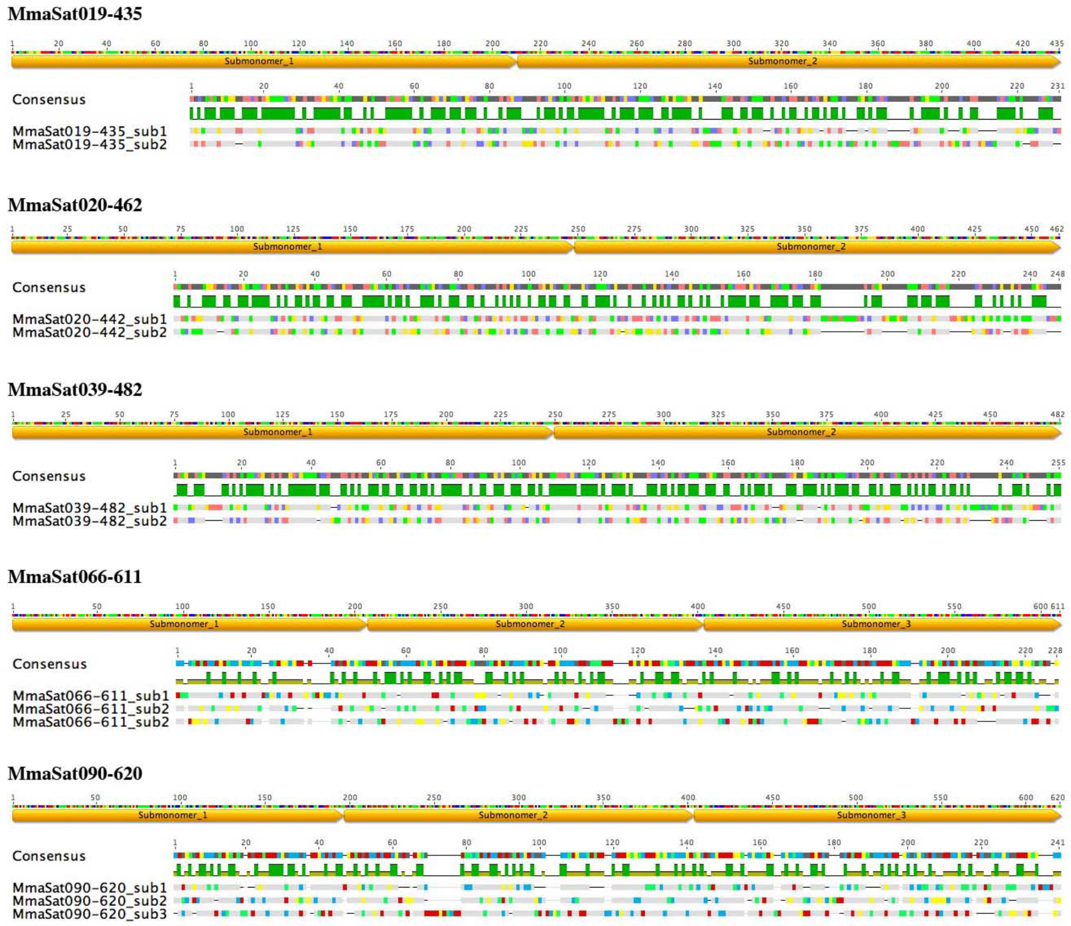

Supplementary Figure S4: Alignment of the five satDNA families included in MmaSF4. Yellow arrows indicate subrepeats.

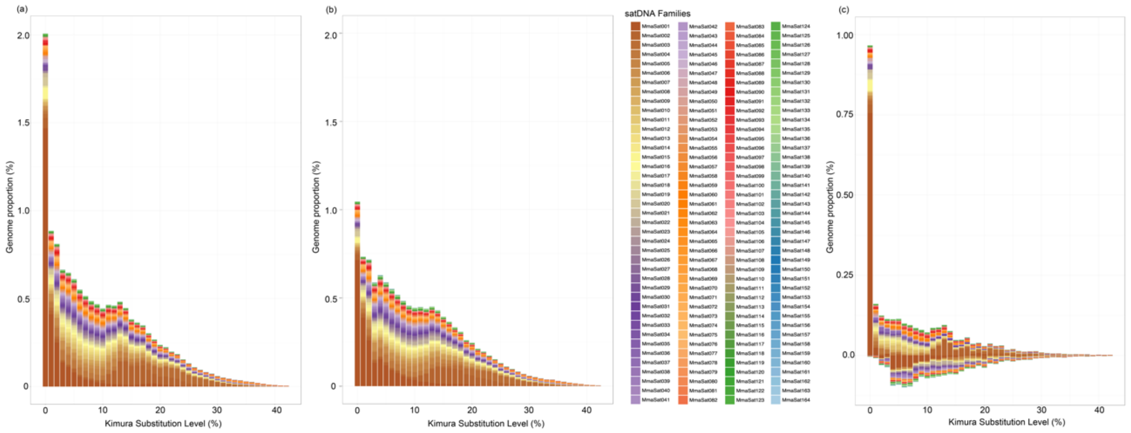

Supplementary Figure S5: Repeat landscapes showing the abundance and divergence profiles for all satDNAs identified in the *M. macrocephalus* female (a) and male (b) specimens, and a subtractive repeat landscape that was obtained by calculating the differences between the male and female counts, which indicates the enrichment of several satDNAs in the female sample that are putatively located on the W chromosome (c).

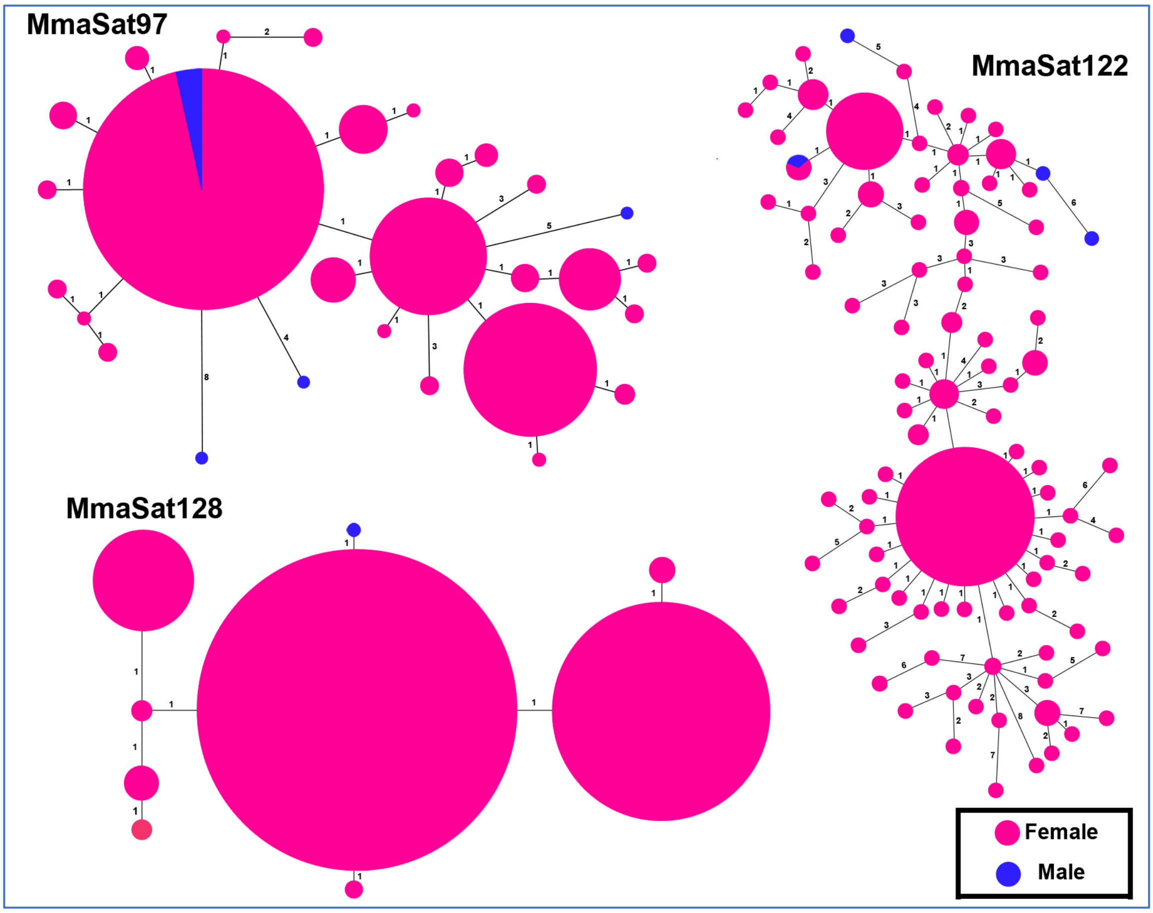

Supplementary Figure S6: Minimum spanning trees (MSTs) showing the relationships between the different haplotypes of MmaSat97, MmaSat122 and MmaSat128 obtained from Illumina reads from males (blue) and females (pink). The diameter of the circles is proportional to their abundance and the numbers represent the number of mutational steps.

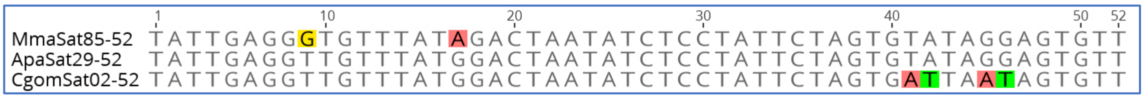

Supplementary Figure S7: Nucleotide alignment between MmaSat85-52 and its respective homologues in *Characidium gomesi* (CgoSat02-52) and *Astyanax paranae* (ApaSat29-52).

Table S1: Repeat unit length (RUL, in nt), A + T content (%), number of variants (V), abundance (% of the genome) in females (F) and males (M) and divergence (%) in females (F) and males (M) of all satDNA families and superfamilies (SF), each showing its own quotient between female and male abundance values (F/M). Asterisks indicate the satDNA families mapped through FISH.

SF	satDNA	RUL	A + T	V	Abundance (%)		Divergence		F/M
					Female	Male	Female	Male	
	MmaSat001-566	566	56.9	2	2.7828955	2.2219752	2.33	3.57	1.25
	MmaSat002-1824	1824	54.6	1	1.2957467	1.0811764	15.2	15.89	1.20
	MmaSat003-1969	1969	54.2	1	0.7739933	0.6412589	21.83	22.36	1.21
	MmaSat004-20	20	50	3	0.5449692	0.5126925	7.77	8.84	1.06
	MmaSat005-1201	1201	59.7	1	0.4234171	0.4233456	11.75	12.6	1.00
	MmaSat006-36	36	66.7	1	0.3554307	0.3698559	6.17	6.6	0.96
	MmaSat007-42	42	54.8	3	0.2955625	0.3100578	15.72	16.5	0.95
	MmaSat008-45	45	53.3	4	0.2940894	0.2094737	10.25	10.48	1.40
1	MmaSat009-53*	53	58.5	5	0.2515902	0.1372152	9.15	10.23	1.83
2	MmaSat010-236	236	63.6	12	0.2355172	0.2686966	8.13	10	0.88
	MmaSat011-37	37	56.8	1	0.2251984	0.1653774	7.08	7.41	1.36
	MmaSat012-50	50	54	4	0.2065817	0.1813352	13.11	14	1.14
	MmaSat013-45	45	44.4	4	0.1870465	0.1380625	9.96	10.85	1.35
	MmaSat014-1878	1878	60.8	1	0.1741413	0.1456353	7.91	10.11	1.20
8	MmaSat015-39	39	56.4	14	0.1672695	0.1719599	12.55	13.33	0.97
	MmaSat016-51	51	80.4	2	0.1323421	0.1258244	5.31	5.86	1.05
	MmaSat017-72	72	57.9	6	0.1280005	0.0563025	9.01	9.7	2.27
	MmaSat018-62	62	71	4	0.126233	0.1323584	5.89	6.93	0.95
2	MmaSat019-435	435	60.9	1	0.1192614	0.1018148	-0.65	0.01	1.17
2	MmaSat020-442	442	61.1	2	0.1155918	0.1091648	12.41	12.32	1.06
	MmaSat021-27	27	44.4	10	0.1129086	0.2035064	12.83	13.58	0.55
	MmaSat022-41	41	58.5	2	0.1117997	0.085009	9.22	10.26	1.32
	MmaSat023-17	17	58.8	2	0.1067554	0.1024796	17.37	15.66	1.04
	MmaSat024-1045	1045	58.2	1	0.1047098	0.1285669	8.16	8.9	0.81
	MmaSat025-22	22	59.1	1	0.1037866	0.0757158	9.6	10.6	1.37
	MmaSat026-25	25	60	4	0.0948755	0.129752	9.92	10.2	0.73
	MmaSat027-67	67	62.7	4	0.0900267	0.0879088	13.32	13.94	1.02
3	MmaSat028-220	220	51.4	1	0.0893837	0.0699632	5.49	6.48	1.28
	MmaSat029-32*	32	46.9	8	0.0846975	0.0503739	14.12	15.34	1.68
	MmaSat030-38	38	47.4	28	0.082197	0.0612807	16.11	16.8	1.34
	MmaSat031-697	697	54.2	1	0.0792113	0.1005833	6.18	7.15	0.79
11	MmaSat032-33	33	57.6	2	0.0791761	0.093751	8.93	9.89	0.84
	MmaSat033-49	49	55.1	5	0.0777897	0.0793178	10.81	11.45	0.98
	MmaSat034-63	63	60.3	15	0.0708584	0.0662731	8.82	9.71	1.07
	MmaSat035-41	41	53.8	7	0.0685827	0.0609549	14.48	15.09	1.13
	MmaSat036-74*	74	62.2	1	0.0683419	0.000202	6.42	26.04	338.55
	MmaSat037-85	85	64.7	1	0.0672261	0.1102712	6.07	7.01	0.61

9	MmaSat038-54	54	63	2	0.0670939	0.0823434	10.49	11.6	0.81
2	MmaSat039-482	482	62.4	1	0.0650824	0.0925934	7.76	7.36	0.70
	MmaSat040-34	34	67.6	1	0.0637776	0.0865696	8.77	8.82	0.74
	MmaSat041-29	29	51.7	1	0.0634877	0.0502377	15.58	17.62	1.26
12	MmaSat042-67	67	55.2	2	0.0631669	0.058297	12.46	12.8	1.08
2	MmaSat043-247	247	62.3	1	0.0614941	0.0673189	10.7	9.83	0.91
	MmaSat044-5	5	50	1	0.0612175	0.0571754	20.61	22.76	1.07
	MmaSat045-1140	1140	49.6	1	0.0599637	0.0618178	3.93	5.21	0.97
	MmaSat046-37	37	64.9	2	0.0595762	0.0509323	10.48	10.86	1.17
	MmaSat047-177	177	66.1	9	0.0584642	0.0705339	12.19	12.7	0.83
	MmaSat048-1298*	1298	49.2	1	0.0583764	0.0365778	6.3	7.51	1.60
	MmaSat049-1709	1709	52.8	1	0.0582143	0.0536612	4.87	6.48	1.08
2	MmaSat050-246	246	61	4	0.0503393	0.0542129	9.86	10.35	0.93
	MmaSat051-30	30	46.7	2	0.0493721	0.0466131	10.41	11.6	1.06
7	MmaSat052-47	47	46.8	1	0.0478542	0.0415941	11.36	12.78	1.15
	MmaSat053-1053	1053	54.6	1	0.0478078	0.0458397	4.29	5.04	1.04
	MmaSat054-16	16	62.5	3	0.047304	0.032146	10.7	11.91	1.47
2	MmaSat055-196	196	65.3	1	0.0472614	0.0433075	22.59	24.01	1.09
	MmaSat056-35	35	51.4	3	0.0465856	0.0575559	12.49	14.15	0.81
	MmaSat057-30	30	53.3	3	0.0462878	0.0559884	18.03	18.04	0.83
15	MmaSat058-71*	71	49.3	3	0.0444104	0.00608	9.57	9.95	7.30
16	MmaSat059-40	40	55	7	0.0440792	0.045836	13.3	13.89	0.96
	MmaSat060-1683	1683	46.7	1	0.0429138	0.046064	4.34	4.77	0.93
8	MmaSat061-33*	33	45.5	1	0.0428973	0.00878	10.86	12.19	4.89
	MmaSat062-61	61	60.7	5	0.0418853	0.04072	10.75	11.75	1.03
13	MmaSat063-47*	47	42.6	7	0.0413418	0.000943	12.26	20.11	43.82
2	MmaSat064-235	235	63	4	0.0412768	0.0565465	19.94	17.26	0.73
2	MmaSat065-246	246	62.2	3	0.0404248	0.0333543	8.64	10.62	1.21
2	MmaSat066-611	611	61.2	1	0.0400215	0.035666	8.54	8.71	1.12
2	MmaSat067-217	217	61.2	2	0.0398557	0.038925	14.22	15.93	1.02
2	MmaSat068-353	353	62	3	0.038825	0.041121	12.17	13.4	0.94
17	MmaSat069-42	42	52.4	10	0.0385085	0.036769	10.9	10.89	1.05
	MmaSat070-44	44	52.3	5	0.038417	0.0267182	12.37	12.95	1.44
	MmaSat071-37	37	59.5	5	0.0382826	0.0337228	10.59	11.56	1.14
	MmaSat072-1074	1074	48	1	0.0378197	0.0423626	3.8	5.21	0.89
	MmaSat073-90	90	70	1	0.0370268	0.0452805	7.16	8.07	0.82
2	MmaSat074-246	246	61.4	4	0.0367916	0.0456733	5.8	5.49	0.81
2	MmaSat075-250	250	62.4	3	0.0354559	0.0377101	16.87	19.45	0.94
	MmaSat076-58	58	43.1	2	0.0347111	0.0293241	9.2	9.53	1.18
6	MmaSat077-412	412	58.3	2	0.0346631	0.0306705	5.85	7.32	1.13
	MmaSat078-31	31	45.2	2	0.033754	0.0269558	12.67	13.73	1.25
	MmaSat079-38	38	84.2	2	0.0332361	0.0375719	6.06	6.46	0.88
	MmaSat080-29	29	58.6	4	0.0332255	0.027907	13.83	14.74	1.19

10	MmaSat081-135	135	40.7	1	0.0329628	0.0208064	14.8	15.53	1.50
	MmaSat082-626	626	53.2	1	0.0319518	0.0455395	7.08	8.24	0.70
	MmaSat083-58	58	67.2	2	0.031846	0.0223408	6.89	6.9	1.43
	MmaSat084-65	65	64.6	4	0.0309865	0.0300537	11.14	12.25	1.03
	MmaSat085-52	52	67.3	1	0.0309718	0.0352903	15.26	17.01	0.88
	MmaSat086-42	42	47.6	3	0.0308068	0.0228899	12.83	12.93	1.35
2	MmaSat087-248	248	64.5	1	0.0306532	0.0378751	13.38	13.23	0.81
	MmaSat088-1031	1031	44.7	1	0.030538	0.0236998	5.72	7.52	1.29
2	MmaSat089-234	234	64.1	2	0.0302708	0.023811	8.68	10.29	1.27
2	MmaSat090-620	620	64.4	1	0.0298446	0.027355	10.66	9.94	1.09
14	MmaSat091-60	60	56.7	6	0.0292001	0.019625	13.01	14.76	1.49
	MmaSat092-46*	46	54.3	11	0.0289438	0.00369	14.74	33.82	7.85
	MmaSat093-953	953	64.2	1	0.0287561	0.0343241	2.67	3.73	0.84
	MmaSat094-450	450	47.3	2	0.028475	0.0286817	6.65	8.03	0.99
	MmaSat095-41	41	68.3	2	0.0259822	0.0246235	8.61	11.26	1.06
	MmaSat096-47	47	57.4	3	0.0250987	0.0174162	13.27	9.89	1.44
	MmaSat097-39*	39	53.8	3	0.0243614	0.000274	10.39	14.26	88.87
	MmaSat098-37*	37	45.9	7	0.024099	0.0149463	13.69	15.41	1.61
	MmaSat099-31*	31	61.3	5	0.0234336	0.0131652	10.89	11.75	1.78
	MmaSat100-60	60	58.3	4	0.0230059	0.0226877	11.29	10.99	1.01
	MmaSat101-454	454	55.3	1	0.0217928	0.025184	6.49	8.16	0.87
	MmaSat102-330	330	53.6	1	0.0196101	0.0304302	7.87	8.68	0.64
2	MmaSat103-324	324	63	3	0.0185744	0.0139772	1.13	-0.01	1.33
	MmaSat104-752	752	57.2	1	0.0183448	0.019529	7.22	7.37	0.94
	MmaSat105-564	564	60.8	1	0.0178615	0.0222973	12.36	13.57	0.80
2	MmaSat106-282	282	63.1	1	0.0178213	0.0151652	6.4	10.5	1.18
	MmaSat107-44*	44	54.6	1	0.0177503	0.0108991	5.84	7.03	1.63
2	MmaSat108-295*	295	60.3	1	0.0176559	0.0117116	6.92	9.63	1.51
2	MmaSat109-210	210	61.4	1	0.0171549	0.0174661	9.02	12.69	0.98
	MmaSat110-1126	1126	55.8	1	0.0171146	0.0159967	11.92	12.23	1.07
	MmaSat111-33*	33	57.6	3	0.0170218	0.00486	16.72	30.69	3.50
3	MmaSat112-387	387	59.4	2	0.0166205	0.0227629	7.49	7.11	0.73
4	MmaSat113-52*	52	61.5	5	0.0166109	0.00126	12.05	18.25	13.18
	MmaSat114-211	211	67.3	4	0.0157794	0.0287923	15.04	15.94	0.55
7	MmaSat115-41	41	58.5	3	0.0154695	0.0227717	13.31	13.07	0.68
	MmaSat116-1310	1310	62.7	1	0.0154613	0.0175173	11.98	12.13	0.88
	MmaSat117-44	44	61.4	12	0.0151687	0.0219488	10.07	9.55	0.69
5	MmaSat118-66*	66	68.2	1	0.0150426	0.00952	9.16	10.05	1.58
	MmaSat119-70	70	54.3	2	0.0144711	0.0123729	8.85	9.93	1.17
8	MmaSat120-31	31	61.3	2	0.014454	0.0119471	11.49	11.77	1.21
	MmaSat121-45	45	64.4	14	0.0131588	0.00893	11.81	12.63	1.47
	MmaSat122-54*	54	57.4	6	0.0130871	0.000266	9.96	16.68	49.17
	MmaSat123-152	152	55.9	7	0.0130834	0.0143146	8.6	9.5	0.91

2	MmaSat124-237	237	62	1	0.0123884	0.0174618	-3.59	-2.53	0.71
	MmaSat125-1261	1261	54.9	1	0.0119073	0.0086	4.46	5.56	1.38
	MmaSat126-178	178	68	4	0.011842	0.0282457	11.86	11.97	0.42
	MmaSat127-42*	42	64.3	6	0.0114881	0.000682	10.8	25.76	16.85
	MmaSat128-38*	38	57.9	4	0.0110087	0.000373	9.47	15.14	29.53
	MmaSat129-36	36	58.3	9	0.0106561	0.0129946	12.47	14.28	0.82
	MmaSat130-54	54	61.1	4	0.0104615	0.00749	9.79	12.52	1.40
9	MmaSat131-49	49	73.5	1	0.00978	0.00996	19.73	19.08	0.98
10	MmaSat132-121	121	52.9	1	0.00943	0.00654	6.97	7.18	1.44
	MmaSat133-69	69	66.7	1	0.00935	0.0156122	12.41	12.13	0.60
	MmaSat134-29	29	79.3	19	0.00894	0.0256614	16.66	16.28	0.35
	MmaSat135-69	69	72.5	10	0.00856	0.0295993	10.6	9.65	0.29
2	MmaSat136-245	245	62	1	0.00797	0.00895	-0.28	0.01	0.89
	MmaSat137-42	42	64.3	2	0.00776	0.0122588	9.54	8.64	0.63
	MmaSat138-17	17	58.8	1	0.00547	0.0211971	9.62	6.16	0.26
4	MmaSat139-47*	47	70.2	1	0.00503	0.000126	20.27	20.93	39.95
6	MmaSat140-330	330	56.1	1	0.00479	0.00467	5.26	7.04	1.03
12	MmaSat141-62	62	66.1	4	0.00442	0.0145375	12.77	9.96	0.30
	MmaSat142-16	16	56.2	1	0.00405	0.00334	12.8	13.13	1.21
11	MmaSat143-27	27	63	1	0.00404	0.00325	16.05	13.79	1.24
8	MmaSat144-16	16	50	1	0.0026	0.00218	25.1	23.12	1.20
12	MmaSat145-67*	67	55.2	1	0.00111	0.00037	10.57	10.84	2.99
11	MmaSat146-33	33	60.6	1	0.00103	0.000813	28.95	28.66	1.26
	MmaSat147-26	26	53.8	1	0.000857	0.00121	23.16	21.7	0.71
13	MmaSat148-31	31	45.2	1	0.000812	0.000329	18.17	35.72	2.47
7	MmaSat149-48	48	50	1	0.000774	0.00083	22.11	23.89	0.93
8	MmaSat150-31*	31	58.1	1	0.000685	0.000319	15.86	21.31	2.15
8	MmaSat151-33*	33	48.5	1	0.000646	0.000158	26.07	32.66	4.09
	MmaSat152-31*	31	61.3	1	0.000413	0.000211	29.91	31.53	1.96
7	MmaSat153-40*	40	65	1	0.000377	0.0000733	26.6	15.54	5.14
	MmaSat154-30*	30	43.3	1	0.000305	0.000181	25.44	32.57	1.69
15	MmaSat155-71*	71	50.7	1	0.000152		15.94	-	-
1	MmaSat156-53	53	50.9	1	0.000128	0.000175	23.78	27.19	0.73
14	MmaSat157-60	60	58.3	1	0.000124	0.000103	24.55	26.31	1.20
13	MmaSat158-39*	39	41	1	0.0000928		26.05	-	-
	MmaSat159-37	37	29.7	1	0.0000437	0.000138	24.8	20.57	0.32
	MmaSat160-29	29	48.3	1	0.000032	0.0000909	13.52	28.1	0.35
16	MmaSat161-40	40	47.5	1	0.00000693	0.0000821	37.05	28.83	0.08
	MmaSat162-48*	48	47.9	1	0.0000032		35.77	-	-
17	MmaSat163-38	38	39.5	1	0.0000024	0.000132	18.39	22.22	0.02
5	MmaSat164-67	67	68.7	1	0.00000187	0.0000221	22.84	29.11	0.08
				**513**	**13.4706184**	**11.9974773**			

Table S2: List of designed primers in this study

satDNA	Primer F (5'-3')	Primer R (5'-3')
MmaSat009-53	CAACACCAGTACCTGACCT	CATTGCTGTGAGGATTTGAT
MmaSat017-72	CTTACTGAGTTAATACAGCGC	TGGTGTTAATGATCAGATTA
MmaSat029-32	GGTCTGATGGAACAACTGCCC	ACCACACAGGATGGGCAGTT
MmaSat036-74	ACCTTGAAAGTGTCTGTCCA	CAATAGTCCCCAAATACAGTTT
MmaSat048-1298	AAACCCACCCAGACGTGTTT	TCGGCCTGCTTCGTTTAAGT
MmaSat058-71	TCACCGCTACGAGTCCTTTC	GACTCTGTGTTAAGCAGGCT
MmaSat061-33	TCCTCACTCACCAACACACG	CAGGTATGCTGAGCGTGTG
MmaSat063-47	TGTTCCTCAGCGACTCTGGA	AGGACCGGCACTAAATCAGG
MmaSat097-39	AACATTCACTCTTACAACCA	GTAGGGTTGAGCTGTGAGC
MmaSat098-37	AGCCTCCAGGACACTCAG	TTGGGACTCTGTGCTGATG
MmaSat099-31	CAACAGCAACTACACATATC	TTGATGAAAACACAGATAT
MmaSat092-46	CGGAATAGAAAACCAGCTA	TGTTTGAGCTGCATTATAC
MmaSat107-44	ACACTAGAGCAGTTAGCTTT	ATACACACATTAGCTCCGG
MmaSat108-295	TAGAACCGTCAGAACCGCTG	GCCCGTCTGCTGCATAGTAA
MmaSat111-33	CTGAGTTGAGTTGTGAGTCA	CAGACTAGCCAATTATTGAC
MmaSat113-52	GGAAGTTTACTGTGGTTACT	TAGTTTAGGAAGGGTAACAG
MmaSat118-66	CAGTTACTCAGTACATGTAC	AGTTACTACTCACCTCTGTA
MmaSat122-54	GTAATCTGGTTTATTTCTGTAG	GAAAAGCAGAGACGCTATGT
MmaSat127-42	TCATATCTCTGAACAGGAGG	TTTACCTATTTGGAGTTATGC
MmaSat128-38	CCTTTCTCTCTCCACATA	GATAGGAATCACTGACTTA
MmaSat139-47	GGAAGTTTACTGTGGTTACT	TAGTTTAGGAAGGGTAACAG
MmaSat145-67	GTCAGAACTGTACGTCCACC	AGAACAGCTTTTAGAGGTGG
MmaSat150-31	CATTCCTTTATCATCCGG	ATGAAGGAACACTGTTCCG
MmaSat151-33	TGTCCACACTCATCAAAGA	GGACAGGTATGCTGCTGGC
MmaSat152-31	GGTCTGATGGAACAACTGCCC	ACCACACAGGATGGGCAGTT
MmaSat153-40	CATGACAATCAGACTAAACT	TAAAGCTCAGGAGATATATC
MmaSat154-30	CTCGCTCAAACCAACTCCAG	AGATGGTGTGTGGGCTGGA
MmaSat155-71	GCGAATCTGGACCGGATG	GATTCGCTGAGGACGGGCG
MmaSat158-39	CCTTTCCTACGACTAATCA	CCTTTCCTACGACTAATCA
MmaSat162-48	CCCTTCGCTCCCTACACA	CTAACATAATGGCATACCCC
